# Intercellular exchange of Wnt ligands reduces cell population heterogeneity during embryogenesis

**DOI:** 10.1038/s41467-023-37350-x

**Published:** 2023-04-06

**Authors:** Yudai Hatakeyama, Nen Saito, Yusuke Mii, Ritsuko Takada, Takuma Shinozuka, Tatsuya Takemoto, Honda Naoki, Shinji Takada

**Affiliations:** 1 Exploratory Research Center on Life and Living Systems (ExCELLS), National Institutes of Natural Sciences, 5-1 Higashiyama, Myodaiji-cho, Okazaki, Aichi 444-8787 Japan; 2 National Institute for Basic Biology, National Institutes of Natural Sciences, 5-1 Higashiyama, Myodaiji-cho, Okazaki, Aichi 444-8787 Japan; 3 The Graduate University for Advanced Studies (SOKENDAI), 5-1 Higashiyama, Myodaiji-cho, Okazaki, Aichi 444-8787 Japan; 4 Graduate School of Integrated Sciences for Life, Hiroshima University, 1-3-2 Kagamiyama, Higashi-Hiroshima, Hiroshima 739-8511 Japan; 5 PREST, Japan Science and Technology Agency (JST), Kawaguchi, Saitama 332-0012 Japan; 6 Nara Institute of Science and Technology, 8916-5 Takayama-cho, Ikoma, Nara, 630-0192 Japan; 7 Institute of Advanced Medical Sciences, Tokushima University, 3-18-5 Kuramoto-cho, Tokushima, Tokushima 770-8503 Japan

**Keywords:** Embryonic induction, Morphogen signalling, Gastrulation

## Abstract

Wnt signaling is required to maintain bipotent progenitors for neural and paraxial mesoderm cells, the neuromesodermal progenitor (NMP) cells that reside in the epiblast and tailbud. Since epiblast/tailbud cells receive Wnt ligands produced by one another, this exchange may average out the heterogeneity of Wnt signaling levels among these cells. Here, we examined this possibility by replacing endogenous Wnt3a with a receptor-fused form that activates signaling in producing cells, but not in neighboring cells. Mutant mouse embryos show a unique phenotype in which maintenance of many NMP cells is impaired, although some cells persist for long periods. The epiblast cell population of these embryos increases heterogeneity in Wnt signaling levels as embryogenesis progresses and are sensitive to retinoic acid, an endogenous antagonist of NMP maintenance. Thus, mutual intercellular exchange of Wnt ligands in the epiblast cell population reduces heterogeneity and achieves robustness to environmental stress.

## Abstract (web summary)

Neuromesodermal progenitor (NMP) cells produce and receive Wnt ligands. Here, the authors show that mutual intercellular exchange of Wnt ligands reduces heterogeneity between NMP cells and makes NMP populations robust to environmental stress.

## Introduction

The number of stem and progenitor cells is tightly controlled during embryogenesis and homeostasis. As the developmental context or external environment surrounding these cells changes, stem and progenitor cell populations respond to these changes, thereby keeping these cells under control. In many cases, secreted signal proteins control the maintenance and differentiation of stem/progenitor cells^1–3^. However, mechanisms by which these proteins contribute to the robustness of these cell populations remain to be determined.

The body axis of vertebrate embryos elongates in an anterior-to-posterior fashion. During this elongation process, cells that constitute tissues in the trunk and tail are continuously generated from progenitor cells^4^. These progenitor cells are found in an area at the posterior end of embryos, termed the epiblast in early embryonic stages and called the tailbud in later stages. Clonal lineage analysis revealed that both neural and paraxial-mesodermal cell types are commonly generated from the same progenitor cells throughout the period of axis elongation^5^. These bipotent progenitor cells are called “neuromesodermal progenitor” (NMP) cells^6–9^. NMP cells appear just before the onset of somitogenesis and are maintained until the conclusion of axis elongation. In mouse embryos, NMPs are located in the caudal lateral epiblast (CLE) posterior to the node-streak border of the primitive streak region and the chordoneural hinge (CNH) of the tailbud^5,10–14^. Population and clonal analyses indicate that these cells behave like stem cells^10,11,15^.

Cell signaling molecules and transcription factors are implicated in the regulation of axis elongation, probably by maintaining NMPs^15–19^. For instance, in the mouse, at least three Wnt ligands are sequentially expressed in the epiblast and tailbud. *Wnt3* expression is first activated in the posterior epiblast at E5.5, followed by *Wnt8a* and *Wnt3a* expression^16–19^. While *Wnt3* and *Wnt8a* expression cease by early somite stage, *Wnt3a* expression continues until E12.5, when tail elongation is almost completed^20^. Along with the expression of these Wnt ligands, a T-box transcription factor, T/Brachyury (Bra), is continuously expressed in the same region from the onset to the end of *Wnt3* expression^21^. Evidence suggests that Wnt signaling and Bra are important for the maintenance of NMPs. Genetic studies of null mutant embryos of *Wnt3a* and *Bra*, showed their importance for axis elongation^16,22^, and lineage tracing of cells that express *Bra* revealed that both neural and paraxial mesoderm cells are derived from *Bra*-expressing cells^23–25^. In addition to NMP maintenance, Wnt signaling and Bra are involved in fate determination between the neural and paraxial-mesodermal lineages^22,26^. Of note, Wnt signaling directly activates *Bra* expression through Tcf transcription factor, while *Bra* is required for *Wnt3a* expression^22,27–29^. Thus, *Wnt* and *Bra* form a positive feedback loop in which each actively regulates the expression of the other in NMP maintenance. Similarly, positive feedback between *wnt8* and *tbxta* (*ntl*), a zebrafish ortholog of *Bra*, has been reported in zebrafish^30–32^. During maintenance of NMPs, activation of Wnt signaling and Bra expression overlap widely in the epiblast and tailbud, including the area where NMPs exists^22^. Thus, some Wnt ligands may act in an autocrine manner in the epiblast and the tailbud, resulting in self-activation of a Wnt/Bra regulatory loop in each cell. On the other hand, given that cells adjoining NMP cells also express Wnt ligands and Bra, paracrine Wnt ligands supplied by neighboring cells may also be involved in NMP maintenance^33,34^.

In this study, to examine the importance of Wnt paracrine function in the maintenance of NMPs, we generated knock-in mouse embryos in which endogenous Wnt3a is replaced with a receptor-fused form that lacks paracrine activity, but maintains autocrine activity. Exacting analysis of Wnt paracrine-deficient embryos revealed the significance of the paracrine signal for the maintenance of the NMP cell population and resilience to stress from surrounding tissue.

## Results

### Lack of paracrine activity in Wnt3a fused with Frizzled

**Fig. 1:**
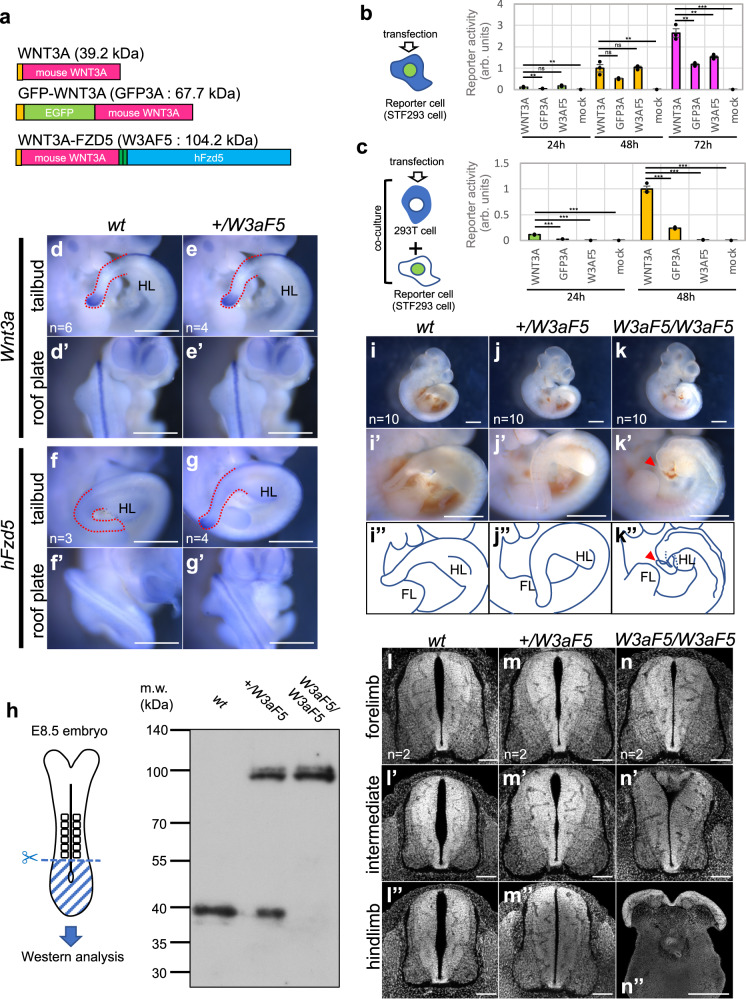
In vitro activity of WNT3A-FZD5 and generation of Wnt3a-Fzd5 knock-in mice. **a** Schematic figure of WNT3A-FZD5, comparing it with WNT3A and GFP-WNT3A. **b**, **c** Wnt signaling activity of each construct shown in **a**. Wnt signaling activity was monitored in HEK293T cells stably expressing the Super TOPFlash reporter (STF293 cells)^36^. In (**b**), Wnt activity was monitored at 24, 48, and 72 h after transfection of each plasmid into STF293 cells. In (**c**), paracrine Wnt activity was monitored in co-cultures of transfected HEK293T cells with STF293 cells at 24 and 48 h after transfection. Units of reporter activity in (**b**) and (**c**) are standardized in activity of WNT3A at 48 h after transfection. Data are presented as mean ± S.D. (*n* = 3 per each condition). Statistical significance was assessed using two-sided Student’s *t* test; ns (not statistically significant, *p*  >  0.05), *p  <  0.05, **p  <  0.01, ***p  <  0.001. In (**b**), WNT3A vs GFP3A *p* = 0.0078 at 24 h, *p* = 0.052 at 48 h, *p* = 0.0021 at 72 h; WNT3A vs W3AF5 *p* = 0.085 at 24 h, *p* = 0.85 at 48 h, *p* = 0.0060 at 72 h; WNT3A vs mock *p* = 0.0014 at 24 h, *p* = 0.0047 at 48 h, *p* < 0.001 at 72 h. **d**–**g** Expression of *Wnt3a-Fzd5* in *Wnt3a-Fzd5* heterozygous (*+/W3aF5*) embryos. Whole-mount in situ hybridization was carried out using probes of mouse *Wnt3a* (**d**, **e**) or human *Fzd5* (**f**, **g**) in wild-type (**d**, **f**) and +*/W3aF5* (**e**, **g**) embryos at E10.5. Images are highlighted on the tailbud (**d**–**g**) and the dorsal neural tube (**d**’, **e**’, **f**’, **g**’). Numbers of stained embryos are indicated by “*n* = “ in the images. **h** Western blotting analysis of proteins prepared from E8.5 embryos with anti-mouse Wnt3a antibody. Two independent experiments were repeated with similar results. **i**–**k** Lateral views of *wt* (**i**), *+/W3aF5* (**j**) and *W3aF5/W3aF5* (**k**) embryos at E10.5. (**i**’), (**j**’), and (**k**’) are magnified images of (**i**), (**j**), and (**k**), respectively. (**i**”), (**j**”), and (**k**”) are drawings of the images of (**i**’), (**j**’), and (**k**’), respectively. **l**–**n** Transverse sections of the neural tube of *wt* (**l**, **l**’, **l**”), *+/W3aF5* (**m**, **m**’, **m**”) and *W3aF5/W3aF5* (**n**, **n**’, **n**”) embryos at E11.5. Sections of DAPI-stained embryos at the forelimb (**l**, **m**, **n**), the intermediate between fore and hindlimb (**l**’, **m**’, **n**’) and the hindlimb (**l**”, **m**”, **n**”) levels are shown. Two embryos were examined for each genotype. Scale bars: 1 mm (**d**–**g**’, **i**–**k**”), 100 μm (**l**–**n**”). HL: hindlimb. FL: forelimb.

To eliminate the paracrine activity of WNT3A, we fused mouse WNT3A to the N-terminus of human FRIZZLED5 via 2 MYC tags (WNT3A-FZD5; Fig. 1a). Activity of WNT3A-FZD5 was examined in cells stably expressing TOPFlash reporter (STF293 cells)^35,36^ in comparison with authentic WNT3A, as well as GFP-fused WNT3A (GFP-WNT3A; Fig. 1a). Activity of GFP-WNT3A was lower than that of authentic WNT3A (Fig. 1b), but sufficient to replace endogenous Wnt3a in vivo^37^. WNT3A-FZD5 activated canonical Wnt signaling to almost the same extent as authentic WNT3A and more strongly than GFP-WNT3A, 48 h after transfection. This activity was nearly saturated after even longer incubation (Fig. 1b). In contrast, whereas Wnt activity was activated in STF293 cells co-cultured with cells expressing intact WNT3A or GFP-WNT3A, almost no activation was detected in co-culture with WNT3A-FZD5-expressing cells (Fig. 1c). Consistent with this result, WNT3A-FZD5 was not detected in the culture supernatant (Supplementary Fig. 1). These results show that as expected, WNT3A-FZD5 possesses sufficient signaling activity, but almost no paracrine activity.

### Impaired axis elongation in *Wnt3a-Fzd5* homozygous embryos

We next generated mouse embryos in which endogenous *Wnt3a* is substituted for *Wnt3a-Fzd5*, using a CRISPR/Cas9-mediated knock-in approach (Supplementary Fig. 2a–f). Mice heterozygous for *Wnt3a-Fzd5* were morphologically normal and fertile (Fig. 1d–g, Supplementary Fig. 2g–k). As expected, *Wnt3a-Fzd5* exhibited an expression pattern identical to that of endogenous *Wnt3a* in these embryos (Fig. 1d–g, Supplementary Fig. 2g–j). In addition, Western blotting analysis revealed that *Wnt3a-Fzd5* heterozygous and homozygous embryos expressed WNT3A-FZD5 proteins at the expense of authentic Wnt3a in the posterior region (Fig. 1h). Thus, *Wnt3a-Fzd5* properly replaced endogenous *Wnt3a*, being expressed in the same spatial pattern as endogenous *Wnt3a*.

While *Wnt3a-Fzd5* heterozygous mice (*+/W3aF5*) showed no obvious abnormality (Fig. 1i, j), *Wnt3a-Fzd5* homozygotes (*W3aF5/W3aF5*), were embryonically lethal and died after E12.5 (Supplementary Fig. 2k). To better understand this puzzling phenotype of *Wnt3a-Fzd5* homozygous embryos, we examined their morphology. While the gross morphology of *Wnt3a-Fzd5* homozygous embryos appeared normal in the anterior trunk, it was highly disorganized posterior to the hindlimbs (Fig. 1i–k). Transverse images of E11.5 embryos stained with DAPI showed that neural tube morphology was gradually disturbed along the anterior-posterior axis in these embryos (Fig. 1l–n). This disruption was evident in the intermediate region between fore- and hindlimbs (Fig. 1n’) and pronounced in the more posterior region, resulting in an open neural tube at the hindlimb level (Fig. 1n”). Interestingly, a thin, kinked tail-like structure was found at the posterior end of these embryos (Fig. 1k: red arrowhead).

**Fig. 2:**
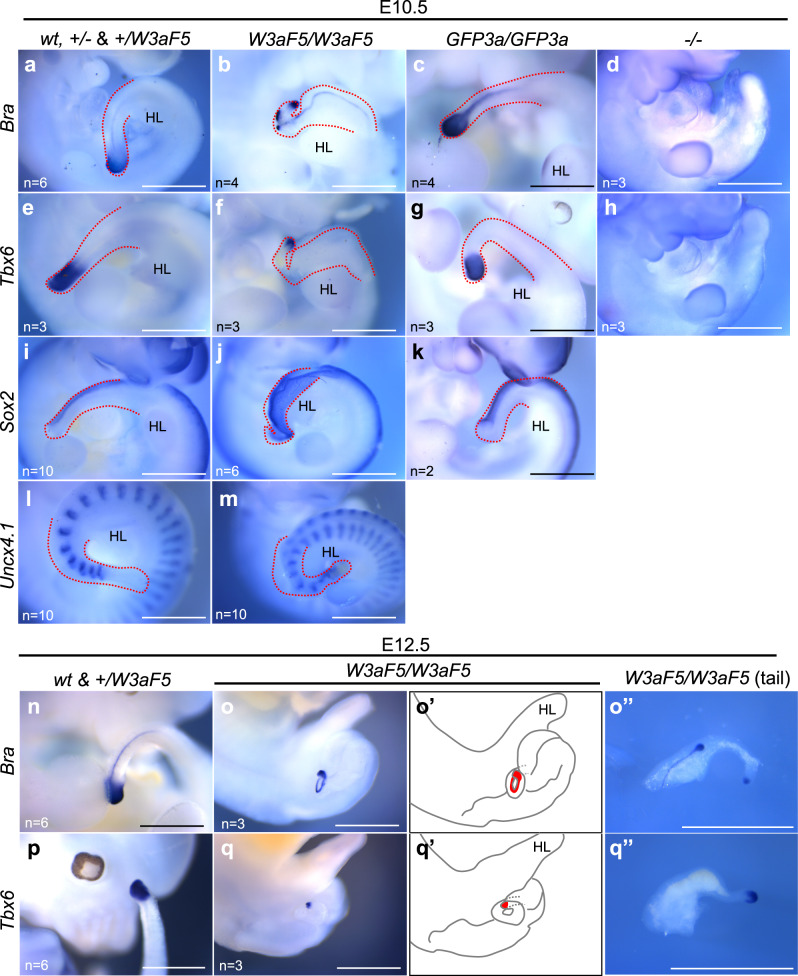
Expression of mesoderm and neural marker genes in *Wnt3a-Fzd5* homozygous embryos. **a**–**m** Expression of mesoderm and neural marker genes in embryos at E10.5. Whole-mount in situ hybridization was carried out using probes of *Bra* (**a**–**d**), *Tbx6* (**e**–**h**), *Sox2* (**i**–**k**), and *Uncx4.1* (**l**, **m**) in *wt, Wnt3a*^+/−^, & *Wnt3a*^*+/Fzd5*^ (**a**, **e**, **i**, **l**), *Wnt3a*^*Fzd5/Fzd5*^ (**b**, **f**, **j**, **m**), *Wnt3a*^*GFP3a/GFP3a*^ (**c**, **g**, **k**), and *Wnt3a*^*−/−*^(**d**, **h**) embryos at E10.5. Red dotted lines indicate tail regions. **n**–**q** Expression of mesoderm and neural marker genes in embryos at E12.5. Whole-mount in situ hybridization was carried out using probes of *Bra* (**n**, **o**), and *Tbx6* (**p**, **q**) in *wt* & *Wnt3a*^*+/Fzd5*^ (**n**, **p**), *Wnt3a*^*Fzd5/Fzd5*^ (**o**, **q**) embryos at E12.5. Tail regions of stained embryos were cut out and shown in (**o**”) and (**q**”). (**o**’) and (**q**’) are drawings of the images of (**o**) and (**q**), respectively. Numbers of stained embryos are indicated by “n = “ in the images. Scale bars: 1 mm. HL: hindlimb.

Whole-mount in situ hybridization analyses also revealed that the neural tube, marked by *Sox2* expression, was morphologically abnormal posterior to the hindlimb at E10.5 in *Wnt3a-Fzd5* homozygous embryos (Fig. 2i, j). Somites, stained with the *Uncx 4.1* probe^38^, were normally formed in the anterior trunk, but their size was reduced posterior to the hindlimb (Fig. 2l, m). Of note, *Brachyury (Bra)*, which is expressed in the tailbud and notochord of normal embryos^39^, was expressed at the tip of the thin and kinked tail, although the number of *Bra*-positive cells was decreased (Fig. 2a, b). In addition, *Tbx6* expression, which is turned on immediately after specification of the paraxial mesoderm lineage^40^, was also detected at this posterior end (Fig. 2e, f). Furthermore, expression of *Bra* and *Tbx6* at the posterior tip was maintained even at E12.5, when tail elongation is nearly arrested in normal embryos (Fig. 2n–q) Thus, in *Wnt3a-Fzd5* homozygous embryos, trunk morphogenesis was disrupted at the hindlimb level, accompanied by a reduction of tailbud size, but differentiation from the remaining progenitor cells in the reduced tailbud appears to be maintained throughout the period of axis elongation.

The phenotype of *Wnt3a-Fzd5* homozygotes was milder than that of *Wnt3a* null mutant embryos, which die around E9.5 with posterior truncation (Fig. 2d, h)^16^. On the other hand, *GFP-Wnt3a* homozygotes, in which the endogenous *Wnt3a* is replaced with a fusion gene encoding GFP-WNT3A shown in Fig. 1, exhibited almost normal elongation of the trunk and tail, except for a slight twist at the tip of the tail (Fig. 2c, g, k; Supplementary Fig. 3)^37^, although the activity of GFP-Wnt3a was lower than the native Wnt3a in the epiblast as in in vitro experiments (Fig. 1b, c; Supplementary Fig. 4). Thus, even though WNT3A-FZD5 has a higher level of signaling activity than GFP-WNT3A, whose activity level is almost sufficient for axis elongation (Fig. 1b, c), *Wnt3a-Fzd5* only partially substitutes for endogenous *Wnt3a* in embryos.

### Development of Wnt(+) progenitors in *Wnt3a-Fzd5* homozygotes

**Fig. 3:**
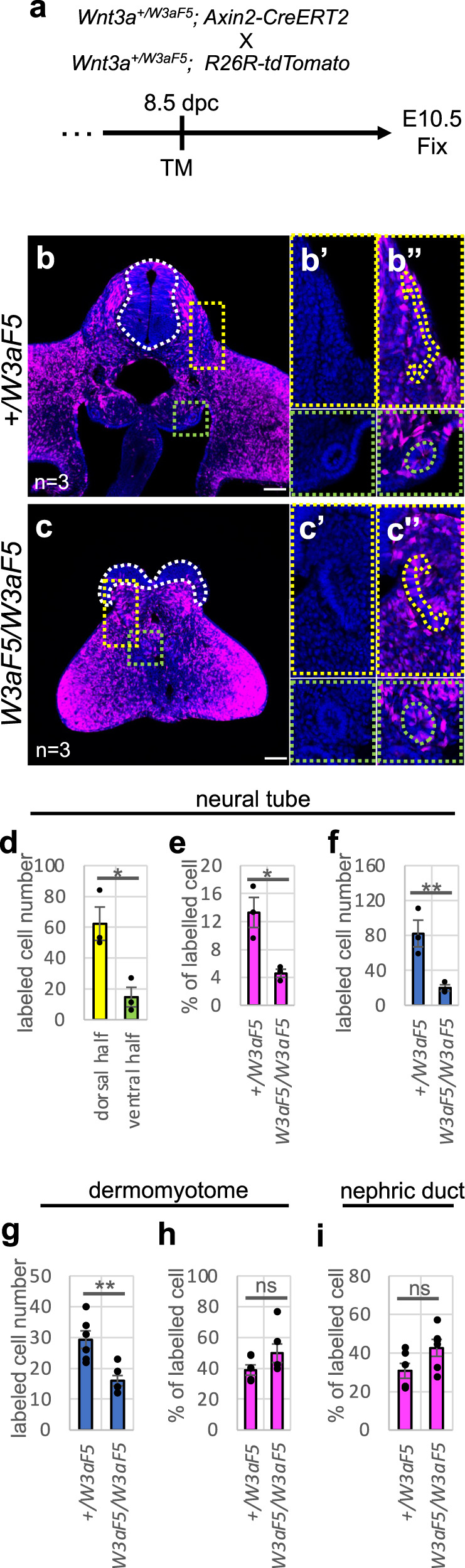
Differentiation of Wnt-positive progenitor cells in the neural tube and somites in *Wnt3a-Fzd5* homozygous embryos. **a** Experimental procedure. Cells activated by Wnt signaling express Axin2-CreERT2, and are eternally labeled by expression of *tdTomato*. Tamoxifen (TM) was injected into pregnant females at 8.5 dpc and embryos were fixed at E10.5. **b**, **c** Distribution of tdTomato-labeled cells at the posterior hindlimb level in *Wnt3a*^*+/Fzd5*^ (**b**) and *Wnt3a*^*Fzd5/Fzd5*^ (**c**) embryos at E10.5. Merged images with DAPI staining are indicated. Magnified images of the dotted yellow and green areas are also indicated without (**b**’, **c**’) or with (**b**”, **c**”) tdTomato fluorescence. Neural tube and dermomyotome, derived from somite, are outlined with white and orange dotted lines, respectively. Squares framed by green dotted lines indicate the area around the nephric duct. **d** The percentage of tdTomato-positive cells in dorsal and ventral neural cells of *Wnt3a*^*+/Fzd5*^. **e**–**i** The percentage of tdTomato-positive cells (**e**, **h**, **i**) and total labeled cell number (**f**, **g**) in neural tube (**e**, **f**), somite (**g**, **h**), and nephric duct (**i**) at the posterior hindlimb level in *Wnt3a*^*+/Fzd5*^ and *Wnt3a*^*Fzd5/Fzd5*^ embryos at E10.5. Numbers or percentages of labeled cells (mean±s.d.) per section are indicated. The photograph (**b**, **c**) and data (**d**–**i**) are representative of three independent biological experiments with similar results. Data are presented as mean ± S.D. Statistical significance was assessed using two-sided Student’s *t* test; ns (not statistically significant, *p*  >  0.05), *p  <  0.05, **p  <  0.01, ***p  <  0.001. *p* = 0.019 in (**d**), *p* = 0.017 in (**e**), *p* = 0.0049 in (**f**), *p* = 0.00083 in (**g**), *p* = 0.051 in (**h**), and *p* = 0.091 in (**i**). Scale bars: 100 μm.

To examine the impact of tailbud reduction on posterior morphogenesis of *Wnt3a-Fzd5* homozygous embryos, we followed cells that showed activated Wnt signaling, because Wnt signaling is activated in progenitor cells in the epiblast and tailbud region. To this end, *Axin2-creERT2* and *floxed tdTomato*^41^ alleles were introduced into *Wnt3a-Fzd5* homozygous embryos. Tamoxifen was injected into pregnant female mice at 7.5 or 8.5 days post-coitus (dpc) and embryos were fixed at E10.5 (Fig. 3a and Supplementary Fig. 5a). In wild-type, and *Wnt3a-Fzd5* heterozygous embryos, labeled cells were detected in most tissues at the hindlimb level, when tamoxifen was injected at 7.5 dpc (Supplementary Fig. 5b–e). In contrast, when tamoxifen was injected at 8.5 dpc, labeled cells were rarely detected in the ventral neural tube at the hindlimb level (Fig. 3b, d; Supplementary Fig. 5d, e), showing that the origin of ventral neural cells loses Wnt signaling after E7.5. Because the proportion of labeled cells was not significantly changed between littermates in tissues except neural tube (Fig. 3e), i.e., somites (Fig. 3h) and the nephric duct (Fig. 3i), we confirmed that cells had once activated Wnt signaling in the epiblast/tailbud at E8.5 were labeled with a similar efficiency in control and *Wnt3a-Fzd5* homozygous embryos. However, comparing with control littermates, the number of labeled cells was specifically reduced in neural tube (Fig. 3f) and somites (Fig. 3g) in *Wnt3a-Fzd5* homozygous embryos. Thus, the number of cells derived from Wnt-positive progenitors at E8.5 was decreased in dorsal neural tube and somites of *Wnt3a-Fzd5* homozygous embryos.

As described above, Wnt3a is expressed in the roof plate of the neural tube, in addition to the epiblast and tailbud (Fig. 1d–g). Thus, it also seems probable that *Wnt3a-Fzd5* expression in the roof plate region causes the morphological abnormality in *Wnt3a-Fzd5* homozygous embryos. To test this possibility, we examined the contribution of *Bra* to this phenotype, because *Bra* interacts specifically with *Wnt3a* in development of the epiblast/tailbud, but not the roof plate. While *Wnt3a-Fzd5* heterozygotes (Supplementary Fig. 6a, d) and *Bra* single heterozygotes (Supplementary Fig. 6b, e) appeared normal, *Wnt3a-Fzd5* and *Bra* compound heterozygous embryos (*Wnt3a*^*+/Fzd5*^*;Bra*^*+/-*^) had open neural tubes and bent tails.(Supplementary Fig. 6c, f). Thus, *Wnt3a-Fzd5* function in the epiblast and tailbud region appears to be involved in the phenotype of *Wnt3a-Fzd5* homozygous embryos.

### *Wnt3a-Fzd5* phenotype due to lack of paracrine activity

Reduction of the Wnt3a signal impairs maintenance of the tailbud, including NMP cells^16,24^. This defect results in truncation of A-P elongation in a manner dependent on Wnt3a activity^42^. To further investigate whether the characteristic phenotype of *Wnt3a-Fzd5* homozygous embryos, including the persistence of small number of tail bud cells, was caused solely by decreased Wnt3a activity, we compared these embryos with *Wnt3a* hypomorphic mutant embryos that exhibit truncation of axial elongation to the same degree as in *Wnt3a-Fzd5* homozygous embryos. To this end, we focused on *Wnt3a*^*vt/-*^ (*vt/-*) embryos, which possess one copy of a hypomorphic (*vt*) allele of *Wnt3a* with reduced *Wnt3a* expression after E8.0 in the tailbud (Supplementary Fig. 4, 7)^42^ and impair trunk development at the hindlimb level (Supplementary Fig.8). In contrast to *Wnt3a-Fzd5* homozygotes, *vt/-* embryos failed to maintain the tailbud, marked by *Bra* and *Tbx6* expression, at E10.5 (Supplementary Fig. 8d–k) and E12.5 (Supplementary Fig. 8i, m). Therefore, the phenotype of *Wnt3a-Fzd5* homozygotes is unique, compared with other *Wnt3a* hypomorphic mutants, and is not simply due to decreased Wnt3a activity.

This characteristic phenotype of *Wnt3a-Fzd5* homozygous embryos was also observed in *Wnt3a*^*Fzd5/-*^ embryos (Supplementary Fig. 9a–c, e–g), whereas *Wnt3a-Fzd5* heterozygous embryos (*Wnt3a*^*+/Fzd5*^) appeared normal, as previously described (Fig. 1j, Supplementary Fig. 9a, e). Thus, *Wnt3a-Fzd5* seems to cause this phenotype in the absence of wild-type *Wnt3a*. Consistently, this phenotype can be rescued depending on the expression level of normal Wnt3a, because the phenotype of *Wnt3a*^*Fzd5/-*^ embryos was partially rescued by replacing the null allele to *vt* (*Wnt3a*^*Fzd5/vt*^; Supplementary Fig. 9d, h). Based on the results of these analyses using various *Wnt3a* mutants, the characteristic phenotype of *Wnt3a-Fzd5* homozygotes is due to some property lost in Wnt3a-Fzd5, most likely paracrine activity.

### NMP cells in *Wnt3a-Fzd5* homozygous embryos

In spite of improper development in the posterior neural tube and somites, our analyses with molecular markers revealed that differentiation of paraxial mesoderm and neural cells was partially maintained in *Wnt3a-Fzd5* homozygous embryos (Fig. 2i–m). Furthermore, the tailbud, marked by expression of *Bra*, was maintained at the posterior tip of the tail of these embryos (Fig. 2a, b, e, f, n–q). These data suggest that a small number of NMP cells persist in *Wnt3a-Fzd5* homozygous embryos.

**Fig. 4:**
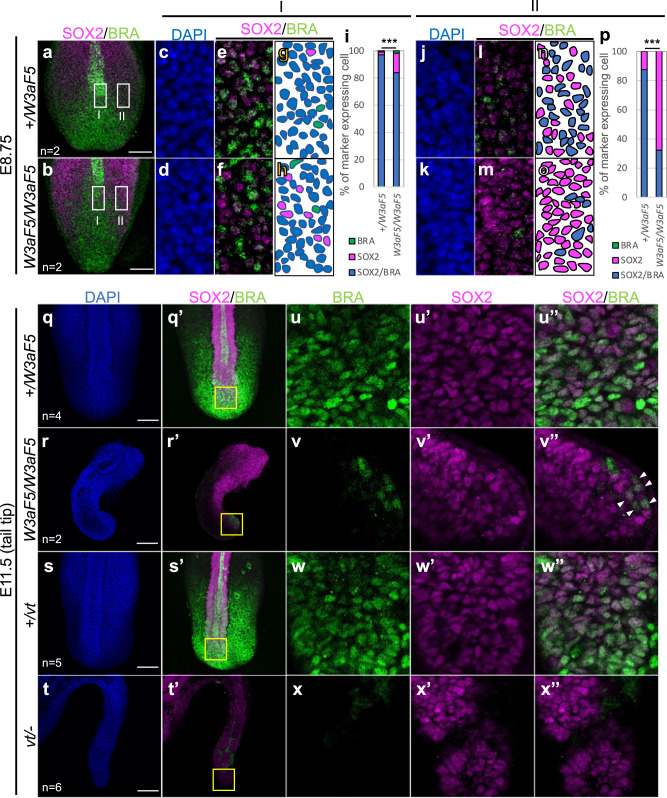
NMP cells in *Wnt3a-Fzd5* homozygous embryos. **a**–**p** Whole-mount staining of *Wnt3a-Fzd5* heterozygous (**a**, **c**, **e**, **g**, **j**, **l**, **n**) and homozygous (**b**, **d**, **f**, **h**, **k**, **m**, **o**) embryos at E8.75. Maximum intensity projection images of posterior ends of embryos stained with anti-SOX2 (magenta) and anti-BRA (green) antibodies are shown in (**a**) and (**b**). To quantify the number of SOX2/BRA double-positive cells, single-plane images of medial (region I) and lateral (region II) regions were analyzed (**c**–**p**). These images contain all cells in the frame regions. While region I contain the node-streak border, region II is set 50 μm away from the side of region I. Images of DAPI staining (blue; **c**, **d**, **j**, **k**), and merged images of staining with anti-SOX2 (magenta) and anti-BRA (green) antibodies (**e**, **f**, **l**, **m**) are shown. Summarized schematic figures (**g**, **h**, **n**, **o**) and diagrams (**i**, **p**) are also shown. In both region I and II, it was confirmed by two-sided κ-square that the population of SOX2/BRA double-positive cells is significantly decreased in *Wnt3a-Fzd5* homozygous embryos (***p < 0.001) . The size of the medial and lateral regions is 50 μm × 100 μm. Two embryos were examined for each genotype. **q**–**x** Whole-mount staining of *Wnt3a*^*+/Fzd5 *^(**q**, **u**), *Wnt3a*^*Fzd5/Fzd5 *^(**r**, **v**), *Wnt3a*^*+/vt *^(**s**, **w**), and *Wnt3a*^*vt/−*^ (**t**, **x**) embryos at E11.5. Maximum intensity projection images of posterior ends of embryos stained with DAPI (blue; **q**–**t**), and with anti-SOX2 (magenta) and anti-BRA (green) antibodies (**q**’–**t**’), are shown. Single-plane images of areas indicated with yellow-lined boxes in (**q**’–**t**’) are magnified in (**u**–**x**), respectively. Images of staining with anti-SOX2 (magenta; **u**–**x**) and anti-BRA (green; **u**’–**x**’) antibodies, as well as merged images (**u**”–**x**”) are shown. The yellow-lined box is a square with one side = 100 µm. Arrowheads in (**v**”) indicate a small number of SOX2/BRA-positive cells. Note that there are no SOX2 and BRA double-positive cells in *Wnt3a*^*vt/− *^(**t**, **x**). The number of stained embryos is indicated by “n = “. Scale bars: 100 μm (**a**, **b**, **q**–**t**).

Since one of the characteristics of NMP cells is the expression of *Bra* and *Sox2*^43^, we compared the number of Bra and Sox2 double-positive cells using immunohistochemistry in *Wnt3a-Fzd5* homozygous embryos and control littermates (Fig. 4a–r, u, v and Supplementary Fig. 10). The number of Bra and Sox2 double-positive cells was reduced at E8.75 in *Wnt3a-Fzd5* homozygous embryos (Fig. 4a–p), but a small number of double-positive cells were still maintained even at E11.5 (Fig. 4q, r, u, v). In contrast, double-positive cells disappeared in *vt/-* embryos at E11.5 (Fig. 4s, w, t, x). These results support the idea that a small number of NMP cells are specifically maintained in *Wnt3a-Fzd5* homozygous embryos, even after trunk development is impaired.

### Wnt signaling activity in *Wnt3a-Fzd5* homozygous embryos

To further investigate the defect of *Wnt3a-Fzd5* homozygous embryos, we directly examined Wnt signaling activity in individual cells in the epiblast. To this end, we utilized the R26 WntVis reporter, the expression of which is driven by heptameric TCF/LEF1 binding sequences combined with a viral minimal promoter in the *Rosa26* locus^44^. This reporter responds in a graded fashion to a wide range of Wnt signal strengths. In addition, histone H2B-GFP protein, used as a fluorescent reporter, facilitates single-cell resolution analysis under confocal microscopy. In this study, the fluorescence of this reporter was measured in individual cells in a photon-counting mode. Epiblast cells in the areas surrounding the node (region 1) and/or those lateral to the node (region 2) were individually analyzed in a single confocal plane.

During the development of mouse epiblast, three Wnt ligands, Wnt3, Wnt8, and Wnt3a, sequentially activate Wnt signaling^16,18,21,45^. Because *Wnt3* and *Wnt8* are expressed prior to *Wnt3a*, Wnt activity was detected even in *Wnt3a* null mutant embryos at the early headfold stage (E7.0)^44^. At this stage, no obvious change in Wnt activity was detected in *Wnt3a-Fzd5* homozygous embryos, as predicted (Supplementary Fig. 11a–e). Then, *Wnt3a* expression was activated, and the Wnt signaling level subsequently increased in both control and *Wnt3a-Fzd5* homozygous embryos (Supplementary Fig. 11f–i), but not in *Wnt3a* null embryos (Supplementary Fig. 11j, u, v), at the late headfold stage (E7.5). Thus, Wnt signaling was properly activated in the initial phase of Wnt3a-dependent activation, even in *Wnt3a-Fzd5* homozygous embryos. Of note, in these stages, the level of the fluorescent reporter differed among epiblast cells in both control and *Wnt3a-Fzd5* homozygous embryos.

**Fig. 5:**
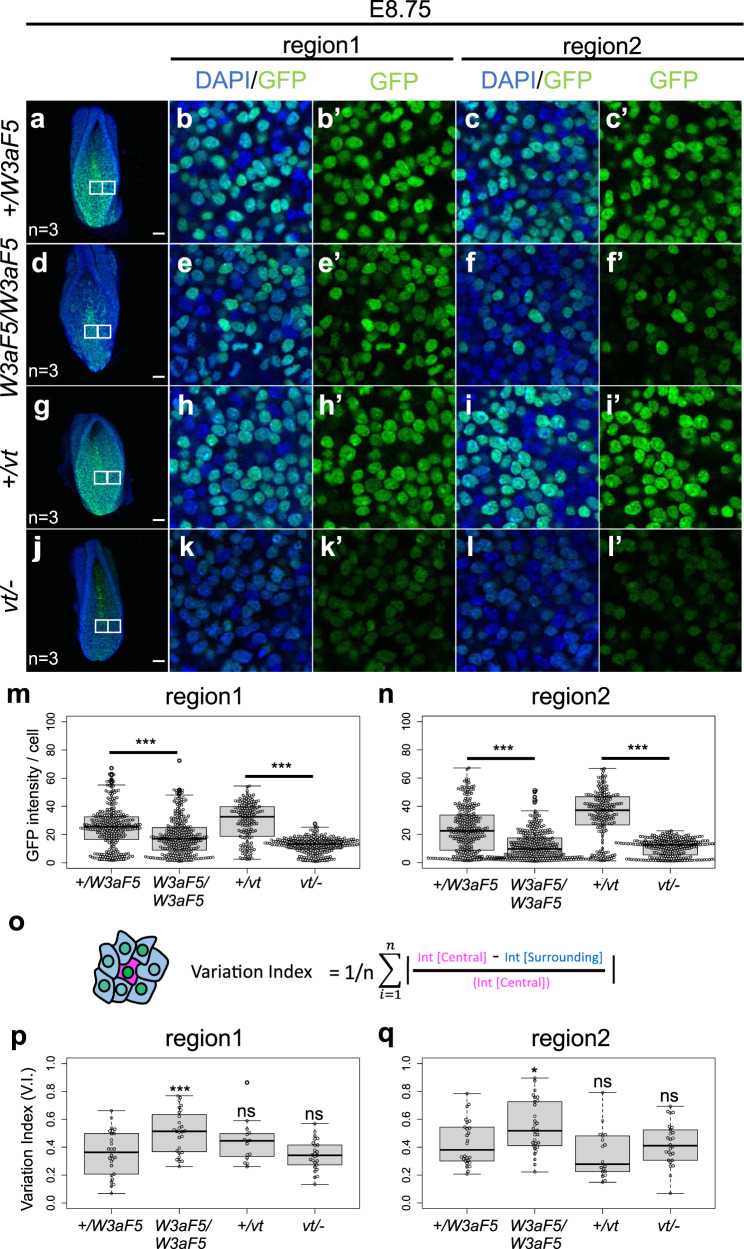
Wnt signaling in the epiblast cell population of *Wnt3a-Fzd5* homozygous embryos. Wnt signaling activity in individual epiblast cells was visualized at E8.75 using mouse embryos carrying an Histone2B-GFP-reporter gene, expression of which is specifically activated by Wnt signaling. Wnt signaling activity was monitored in *Wnt3a-Fzd5* heterozygous (**a**–**c**) and homozygous (**d**–**f**) embryos, as well as in +*/vt* (**g**–**i**) and *vt/-* (**j**–**l**) embryos. In each embryo, magnified images of the areas indicated by boxes are also shown. Magnified images of the region containing the node (region 1) and a lateral region of CLE (region 2) are shown in each genotyped embryo (Areas = 100 × 100 μm). While region 1 contain the node-streak border, region 2 is lateral to region 1. Magnified images were taken in a single confocal plane while others were processed by maximum intensity projection. GFP intensity in individual cells in region 1 and 2 was quantified in each genotyped embryo (**m**, **n**). Three embryos were examined for each genotype except for +*/vt* (2 embryos). Box plots indicate the first and third quartiles and the median. Variation between adjacent cells was also examined (**o**–**q**). Here, we focused on cells that are in the top 10% of Wnt activity in each framed region, and measured the degree of difference in Wnt activity between such cells and surrounding cells as shown in (**o**). The degree of difference, called as “variation index” here, is examined for region 1 (**p**) and 2 (**q**). Each dot in (**p**) and (**q**) indicates variation index around one of cells in the top 10% of Wnt activity. In (**m**), (**n**), (**p**), and (**q**), the middle, upper, and lower box lines represent the maximum, minimum, median and two quartiles of values in each group and whiskers indicate highest and lowest values no greater than 1.5× interquartile range. Differences were assessed for statistical significance using two-sided Student’s *t* test; ***p < 0.001; **p < 0.01; ns, not statistically significant (*p* > 0.05). In (**p**), *+/W3aF5* vs *W3aF5/W3aF5 p* = 0.0010, *+/W3aF5* vs +*/vt p* = 0.12, *+/W3aF5* vs *vt/− p* = 0.69, in (**q**) *+/W3aF5* vs *W3aF5/W3aF5 p* = 0.015, *+/W3aF5* vs +*/vt p* = 0.13, *+/W3aF5* vs *vt/− p* = 0.87. The number of examined embryos is indicated by “n = “. Scale bars: 100 μm.

From the early somite stage (E8.0), Wnt signaling began to be perturbed in *Wnt3a-Fzd5* homozygous embryos. At E8.0, around the 2-3-somite stage, the number of Wnt signaling-positive cells was reduced in the anterior and lateral epiblast regions of *Wnt3a-Fzd5* homozygous embryos (Supplementary Fig. 11l–p). The reduction in Wnt signaling-positive cells was pronounced in both medial (region 1) and lateral (region 2) epiblast regions of *Wnt3a-Fzd5* homozygous embryos at E8.75 (Fig. 5a–f). Considering that lack of paracrine activity can lead to heterogeneity in Wnt signaling between adjacent cells, we focused on the top 10% of cells with strong Wnt signaling to examine the extent to which cells with strong Wnt signaling affect Wnt signaling in surrounding cells. Actually, the heterogeneity of Wnt signaling between neighboring cells, defined as variation index, was significantly increased in *Wnt3a-Fzd5* homozygous embryos at E8.75 (Fig. 5o–q, Supplementary Fig. 11k). The reduction in Wnt signaling-positive cells was enhanced by E9.5, but some Wnt-positive cells remained at the posterior end of *Wnt3a-Fzd5* homozygous embryos (Supplementary Fig. 11w, x). On the other hand, in *vt/-* embryos, the decrease of Wnt signaling started at E8.75 (Fig. 5g–l, Supplementary Fig. 11q–t, y, z). In contrast to *Wnt3a-Fzd5* homozygous embryos, Wnt signaling was almost abolished around E9.5 in epiblast (Supplementary Fig. 11w–z), and the deviation of Wnt-activity in epiblast population was not significantly changed in *vt/-* embryos (Fig. 5o–q).

Taken together, in *Wnt3a-Fzd5* homozygous embryos, reduction of Wnt activity occurred from early somite stage, but a small number of Wnt-positive cells remain longer. Notably, Wnt activity appeared to vary between adjacent cells, even in control embryos, but in *Wnt3a-Fzd5* homozygous embryos this heterogeneity was significantly enhanced (Fig. 5o–q). Probably, the accelerated reduction of Wnt activity in many epiblast cells reduces the number of NMP cells, as well as of neural and somite cells produced by NMP cells in *Wnt3a-Fzd5* homozygous embryos. In contrast, persistent activation of Wnt signaling in the other epiblast cells probably contributes to the maintenance of a small number of NMP cells, resulting in the formation of thin, kinked tails.

### Effect of retinoic acid on *Wnt3a-Fzd5* homozygous embryos

**Fig. 6:**
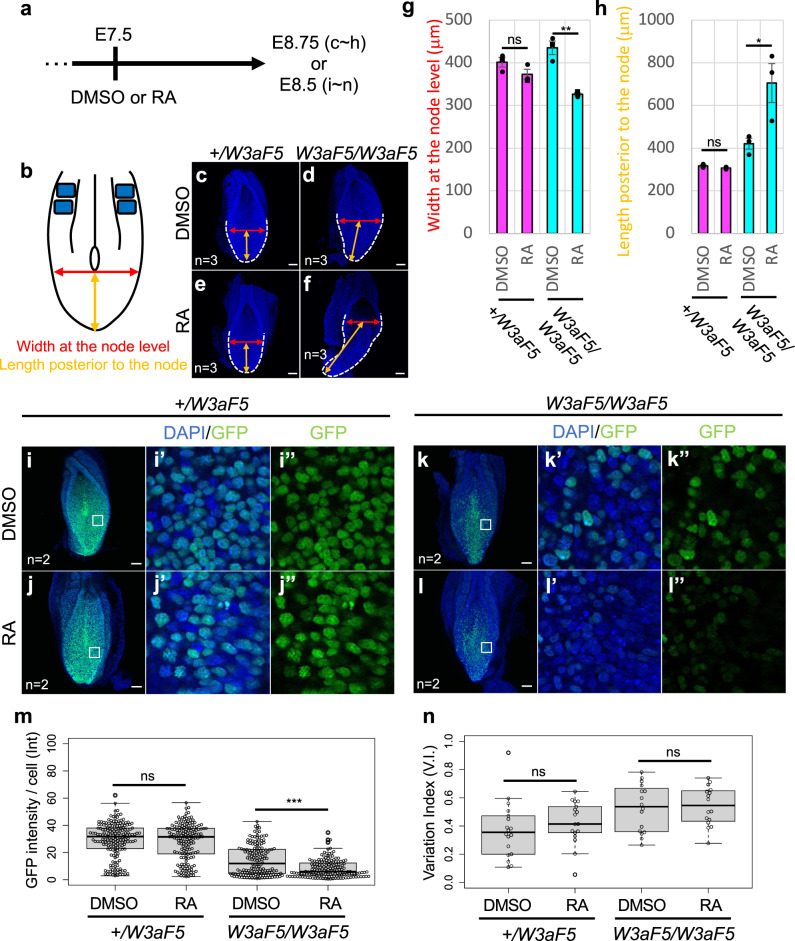
Effect of retinoic acid on the epiblast cell population of *Wnt3a-Fzd5* homozygous embryos. **a, b** Experimental schemes are shown. **c**-**h** Morphological abnormality of RA-treated embryos. Dorsal images of DMSO (**c**, **d**) or RA (**e**, **f**) treated *Wnt3a-Fzd5* heterozygous (**c**, **e**) and homozygous (**d**, **f**) embryos at E8.75 stained with DAPI (blue). Results of quantification of the width of embryos at the node level (**g**) and the length posterior to the node (**h**) in each genotyped embryo are shown. Note that RA treatment enhances the abnormality in gross morphology specifically in *Wnt3a-Fzd5* homozygous embryos. Red arrows indicate the width at the node level while orange arrows indicate the length posterior to the node. Data are presented as mean ± S.D. Statistical significance was assessed using two-sided Student’s *t* test; ns (not statistically significant *p*  >  0.05), *p  <  0.05, **p  <  0.01, ***p  <  0.001. In (**g**) *+/W3aF5* DMSO vs RA *p* = 0.16, *W3aF5 /W3aF5* DMSO vs RA *p* = 0.027, and in (**h**) *+/W3aF5* DMSO vs RA *p* = 0.18, *W3aF5 /W3aF5* DMSO vs RA *p* = 0.047. Scale bars: 100 μm. The number of examined embryos is indicated by “n = “. **i**–**n** Wnt activity in RA-treated embryos. Wnt signaling activity in individual epiblast cells was visualized as shown in Fig. 5. These embryos were treated with RA 7.5 days post-coitus and analyzed at E8.5. Dorsal images, processed by maximum intensity projection of DMSO- (**i**, **k**) or RA- (**j**, **l**) treated *Wnt3a-Fzd5* heterozygous (**i**, **j**) and homozygous (**k**, **l**) embryos are shown. Images of a single confocal plane in the caudal lateral epiblast of these embryos are also indicated with (**i**’-**l**’) and without (**i**”-**l**”) DAPI. The size of these areas is 100 μm × 100 μm and their positions in the epiblast are identical to those of the region 2 in Fig. 5f. GFP intensity in individual cells in (**i**”) - (**l**”) is summarized in (**m**). The “variation index” in Wnt activity, as shown in Fig. 5 is also indicated (**n**). Two embryos were examined for each genotype. In the box and whisker plots, the middle, upper, and lower box lines represent the maximum, minimum, median and two quartiles of values in each group and whiskers indicate highest and lowest values no greater than 1.5× interquartile range. Differences were assessed for statistical significance using a two-sided Student’s *t* test; ns (not statistically significant *p*  >  0.05), *p  <  0.05, **p  <  0.01, ***p  <  0.001. In (**m**), *+/W3aF5* DMSO vs RA *p* = 0.35, *W3aF5 /W3aF5* DMSO vs RA *p* < 0.001, and in (**n**) *+/W3aF5* DMSO vs RA *p* = 0.88, *W3aF5 /W3aF5* DMSO vs RA *p* = 0.57. In (**b**), blue squares indicate somites. Scale bars: 100 μm.

Retinoic acid (RA) antagonizes *Bra* and Wnt signaling at the caudal end of vertebrate embryos during body axis elongation^31^. In mouse embryos, RA signaling is activated in the epiblast, posterior mesoderm, and the node at E7.5, and then gradually disappears from these tissues, with strong activation eventually being limited to somites, anterior PSM and neural tube^13,46–49^. The mutually exclusive spatial pattern of RA signaling and Wnt signaling suggests that progenitor cells in the epiblast/tailbud region, including NMP, are regulated by their antagonism. Thus, we examined whether the resistance of progenitor cells to RA is changed in *Wnt3a-Fzd5* homozygous embryos. Female *Wnt3a-Fzd5* heterozygous mice crossed with *Wnt3a-Fzd5* heterozygous males were treated with RA 7.5 days post-coitus and the effects of RA on the phonotype of mutant embryos were examined. RA treatment specifically enhanced the abnormality in the gross morphology of *Wnt3a-Fzd5* homozygous embryos (Fig. 6a–h). Furthermore, Wnt reporter analysis revealed that RA treatment reduced the average level of Wnt activity, but caused no significant change in heterogeneity, in *Wnt3a-Fzd5* homozygous embryos (Fig. 6i–n). This result suggests that the entire epiblast cell population of *Wnt3a-Fzd5* homozygous embryos is specifically susceptible to RA.

### Study of Wnt paracrine function by mathematical modeling

**Fig. 7:**
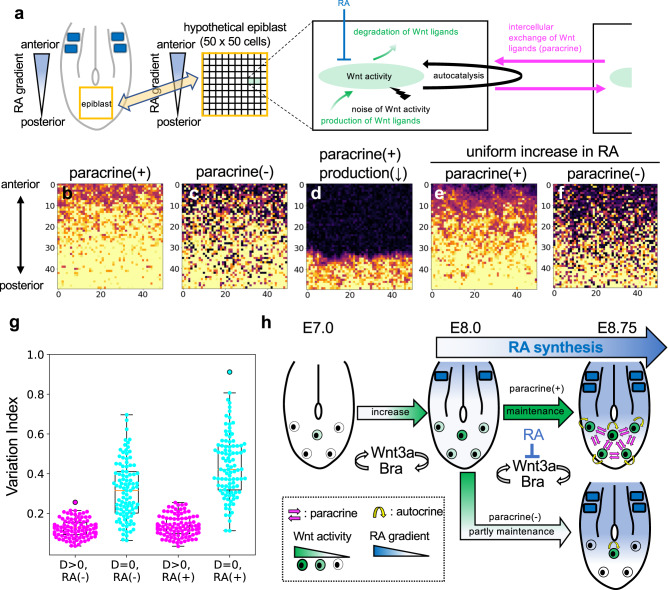
Mathematical model to examine the effect of intercellular communication in cell populations. **a** Schematic diagram showing parameters used in the model. We assumed a virtual space corresponding to the cell sheet of the epiblast. This virtual space is divided into 50 × 50 sections along the antero-posterior and medio-lateral axes. Each section corresponds to a single cell in the epiblast. Wnt activity (W) is determined by parameters such as the rate of production and degradation of Wnt protein, the rate of amplification or reduction by positive feedback, the rate of intercellular exchange of Wnt protein, the rate of inhibition by RA, and fluctuating noise that affects Wnt activity. It is assumed that cell division occurs randomly and that dividing daughter cells are extruded in one section in either the left, right, or anterior direction in a 1:1:2 ratio. **b**–**f** Spatial patterns of Wnt activity in a virtual sheet of cells. Examples of the spatial pattern in the presence (**b**, **d**, **e**) or absence (**c**, **f**) of the paracrine function of Wnt are shown at the same time point (mean division time *t* = 3.00) after the addition of RA (*t* = 0). In the condition of d, the Wnt production rate is reduced (see “Methods”). Spatial patterns of Wnt activity were calculated in the absence (**b**–**d**) and presence (**e**, **f**) of uniformly supplied RA. **g** Variation index was calculated at position 25 and T = TR + 4. Loss of Wnt-mediated cell-to-cell communication increased the variability index (D > 0 vs D = 0 in RA(−)). The variation index was not significantly changed when the RA concentration was uniformly increased in the hypothetical epiblast plane (RA(-) vs RA(+)). In the box and whisker plots, the middle, upper, and lower box lines represent the maximum, minimum, median and two quartiles of values in each group and whiskers indicate highest and lowest values no greater than 1.5× interquartile range. In each condition, 20 independent simulations were performed. **h** Schematic representation showing the effect of Wnt paracrine in the epiblast cell population.

The results described above strongly suggest that lack of paracrine signaling enhances heterogeneity of Wnt activity in the epiblast cell population and that a cell population with such enhanced heterogeneity is more sensitive to antagonists, like RA. Thus, we also tested the validity of these ideas by creating a mathematical model (Fig. 7a–g, Supplementary Fig. 12 and Movie1–5). In this model, spatiotemporal changes in Wnt activity were compared in a hypothetical epiblast plane with and without intercellular exchange of Wnt ligands. The temporal increase or decrease of Wnt activity in each cell is defined by the production rate regulated by autocatalysis, which represents a positive feedback loop of Wnt3a/Bra, in addition to the basic rate of production and degradation of Wnt ligands. The stochastic increase/decrease in Wnt activity is also incorporated as a noise term. In this virtual plane, we assume that each cell divides stochastically and that a newly produced daughter cell locates laterally or anteriorly to the original cell. It is also assumed that an RA gradient from anterior to posterior is imposed at a specific time, which represents anteroposterior diffusion of RA. However, in this virtual space, cells that are aligned along the left-right axis were treated as if there is no difference in their distance from the RA source (Fig. 7a).

By using adjusted parameters, we first simulated spatiotemporal patterns of Wnt activity in the hypothetical epiblast plane under conditions in which intercellular exchange of Wnt ligands is present (Fig. 7b, d, e) and absent (Figs. 7c, f). Wnt activity levels decreased after the addition of RA (*t* > 0 shown in Supplementary Fig. 12 and Movie1, 2) and the number of Wnt-low cells (Fig. 7b, c) and heterogeneity between adjacent cells (variation index; Fig. 7g) increased in the absence of intercellular Wnt exchange. However, a relatively small number of Wnt-high cells remained for a while (until *t* = 4 shown in Fig. 7c Supplementary Fig. 12b and Movie 2). On the other hand, if there was no production except autocatalysis, mimicking the situation of *vt/-* embryos, Wnt-low cells gradually increased and few Wnt-high cell remained (Fig.7d and Supplementary Fig. 12b and Movie 3). These simulations showed that a lack of intercellular Wnt exchange reproduced the spatiotemporal pattern of Wnt activity observed in *Wnt3a-Fzd5* homozygous embryos. Furthermore, we reproduced the increased sensitivity of *Wnt3a-Fzd5* homozygote cells and no significant increase in heterogeneity between adjacent cells, when the RA concentration was uniformly increased in the hypothetical epiblast plane (Fig. 7e–g, and Supplementary Movies 4, 5). Taken together, these simulations based on our mathematical model support the idea that the Wnt paracrine signal reduces heterogeneity in Wnt activity in the epiblast cell population and increases robustness to RA. Finally, we note that the content of Wnt-negative cells was lower in the model than in the experiment, suggesting that some unrevealed mechanism involved in suppression of Wnt signaling in the NMP population may exist.

## Discussion

It is widely believed that secreted signal proteins act on cells surrounding the source cells^50,51^. In contrast, in the epiblast and tailbud, most cells both produce and receive Wnt ligands^24,52–54^. As a result, in this cell population, each cell is activated by Wnt ligands secreted by each other. Thus, in contrast to unidirectional transfer from Wnt-producing cells to receiving cells, Wnt ligands seem to be reciprocally exchanged between cells in the epiblast and tailbud. To understand the biological significance of reciprocal ligand exchange within a cell population, we generated *Wnt3a-Fzd5* homozygous embryos, in which Wnt3a-mediated intercellular communication, or paracrine function, is specifically impaired. In these embryos, the number of cells in which Wnt signaling is strongly activated (Wnt-strong cells) decreases rapidly from the anterior and lateral sides of the epiblast after E8.0, while a small number of Wnt-strong cells, including NMP cells, remain at the posterior end for a long time. Precise examination of *Wnt3a-Fzd5* homozygous embryos and mathematical simulation support a model in which Wnt3a-mediated intercellular communication is crucial for the maintenance of the epiblast cell population, including NMP cells (Fig. 7h).

In epiblast and tailbud regions, including NMPs, Wnt3a-expressing cells also express the transcriptional regulator, Bra^22^. Bra is a direct transcriptional target of the Wnt signaling pathway, whereas Wnt3a expression is also dependent on Bra^22,55^. Thus, Wnt3a and Bra are likely to mutually activate one another, forming a positive feedback regulatory loop. Therefore, once expression levels of *Wnt3a* and *Bra* in the cell begin to increase or decrease, this positive feedback can further amplify the change. On the other hand, it seems plausible that not all epiblast/tailbud cells are identical with respect to Wnt production and degradation rates, the efficiency of feedback amplification, and/or resistance to environmental factors that reduce Wnt activity. Actually, our analysis with EGFP-H2B Wnt reporter shows heterogeneity of epiblast/tailbud cells. Thus, when differences occur between cells in the direction of changes (increase or decrease) in Wnt signaling activity under certain conditions, such differences are amplified by positive feedback, resulting in increased disparity.

In the epiblast of *Wnt3a-Fzd5* homozygotes, the number of cells with little or no Wnt activity rapidly increases from E8.0 in *Wnt3a-Fzd5* homozygous embryos. Probably, this rapid decrease can be explained by Wnt3a/Bra-positive feedback, if we assume that the direction of amplification changes from increase to decrease in these cells after E8.0. One of the candidates to trigger this decrease is an antagonist to the Wnt/Bra positive feedback loop, like RA, as shown below. However, at present, it is uncertain whether RA or some other factor triggers this change in vivo. In contrast, a small, but significant number of cells maintain high Wnt activity for a long time in these embryos. A probable reason for the persistence of Wnt-high cells is that the change of Wnt activity in these cells is below the threshold to trigger a rapid decrease by positive feedback. Actually, our mathematical model, which assumes variation in Wnt activity and positive feedback regulation, produces similar spatial patterns of Wnt activity in *Wnt3a-Fzd5* homozygous cell populations under conditions without Wnt-mediated intercellular communication.

In contrast, in control embryos, including *Wnt3a-Fzd*5 heterozygotes, the number of Wnt3a-positive cells slowly decreased and disappeared around E13.5, when axis elongation was terminated. Probably, in these embryos, Wnt ligands supplied by neighboring Wnt-high cells compensate to some extent for the decrease of Wnt activity in Wnt-low cells. Thus, the exchange of Wnt3a ligands appears to compensate for the rapid decrease in Wnt activity in individual cells. Taken together, positive feedback regulation can amplify heterogeneity among members of the cell population, but our results suggest that sharing of intercellular components of the feedback loop, such as Wnt ligands, counteracts the amplification of this heterogeneity.

In the epiblast of *Wnt3a-Fzd5* homozygotes, Wnt activity rapidly decreases in many cells at E8.0, when several anterior somites are generated. In these embryos, the decrease in Wnt activity was more pronounced anteriorly and laterally in the epiblast (Fig. 5d–f). Of note, while somite and neural cells are generated from NMP cells, the RA-synthesizing enzyme, retinaldehyde dehydrogenase 2 (RALDH2/ALDH1a2), is mainly expressed in somites and lateral plate mesoderm in mouse embryos^56,57^. In addition, a line of evidence has shown that RA signaling antagonizes Wnt/Bra activity gradually from the anterior side of the epiblast and tailbud region in the mouse embryo^31,56,58–60^. Thus, we speculated that the epiblast cell population in *Wnt3a-Fzd5* homozygotes is sensitive to RA stress originating from tissues developed anteriorly and laterally to the epiblast.

Actually, RA treatment reduces Wnt signaling in epiblast cells specifically in *Wnt3a-Fzd5* homozygous embryos, indicating that the epiblast cell population in these embryos is more susceptible to RA. Probably due to a failure to reduce heterogeneity in these embryos, an RA-triggered decrease in Wnt activity may be amplified rapidly via a positive feedback loop in individual cells. Thus, maintaining cooperativity among members of the epiblast/tailbud cell population and reducing the disparity in Wnt signaling among members may render the cell population more resilient to external stress.

It has been shown that intercellular communication within a cell population is critical to regulate cell differentiation. In *Xenopus* gastrulas, muscle progenitor cells communicate with each other as they differentiate. In such a case, more than one hundred *Xenopus* muscle precursor cells transplanted into ectoderm sandwiches can differentiate, while smaller groups and single cells cannot^61–63^. This cell number-dependent differentiation was described as a “community effect.” In this case,  an intercellular interaction among precursor cells seems to be necessary for these cells to differentiate. Theoretical studies have suggested that the positive feedback mediated by intercellular communication is the mechanism underlying this cell number-dependent differentiation^64^.

In this study, we showed that Wnt-mediated intercellular communication is actually involved in maintenance of the cell population in the epiblast/tailbud region. In this case, intercellular exchange of Wnt ligands is important to compensate for the disparity amplified via positive feedback from Wnt3a and Bra. An interesting question is whether a similar molecular network is involved in other events in which a community effect is exerted. Differences in the efficiency of cell signaling or the amplification efficiency of positive feedback loops probably generate differences in the features of cell populations. If this is the case, it will be important in future studies to identify key parameters in the molecular network to produce each of these events.

## Methods

### Mice

Animal care and experiments were performed in accordance with guidelines for animal experimentation of the National Institutes for Natural Sciences. All animal experiments were approved by the Animal Research Committee of National Institutes for Natural Sciences. Mice were maintained in a light- and temperature-controlled room using a 12 h:12 h light:dark cycle at 21 ± 2 °C. *Wnt3a* null and *vt* mice were gifted by Dr. Andrew McMahon. *GFP-Wnt3a* knock-in mice were previously generated by ST^37^. Brachyury null mutant mice were distributed by RIKEN BRC (RBRC00113: C3H/HeSn-*Ttf*/+*tf*). Wnt vis reporter mice were previously generated by TT^44^. C57BL/6 N mice, purchased from Japan SLC and CLEA-Japan, were used to mate with these mutant mice. Mice younger than 60 weeks were mated for collecting embryos. Embryos derived from timed matings were genotyped by PCR with DNA from yolk sacs or embryos. PCR conditions and primer sequences for Wnt3a null^16^ and Wnt vis reporter^44^ mice have been previously described.

### Cell culture and transfection

HEK293T and STF293 cells were provided by Dr. Takeichi (RIKEN) and Dr. Tsukiyama (Hokkaido University), respectively. All cells were routinely tested for the absence of mycoplasma. HEK293T cells and STF293 cells, which are HEK293 cells stably expressing Super 7x TOPFlash^35^, were cultured at 37 °C in a 1:1 mixture of DMEM and Ham’s F12 medium supplemented with 8.3% fetal bovine serum. Cells were inoculated in 24-well plates at a concentration of 1 ×10^5^ cells/well. Plasmids were transfected into HEK 293T cells or STF293 cells using FuGENE6 transfection reagent (Roche). The culture medium was changed 6 h after transfection. At 24, 48, and 72 h after transfection, cells and culture medium were harvested for Western blotting and luciferase reporter assay. In co-culture experiments, HEK 293T cells transfected with each plasmid were collected 24 h after transfection and mixed 1:1 with STF293 cells. These cells were inoculated at a concentration of 1 ×10^5^ cells/well using 24-well plates. The luciferase reporter assay was performed 24 or 48 h after co-culture. Details of Western blotting and the luciferase reporter assay are described below.

### Plasmid construction

To generate plasmid constructs from which Wnt3a fused with human Frizzled 5 (hFzd5) is expressed, a DNA fragment encoding the full length of mouse Wnt3a protein fused to the N-terminus of hFzd5 mediated with 2xMyc tag (TSEQKLISEEDLNEMEQKLISEEDLRS) (Supplementary Fig.1a), was integrated between the ClaI and XbaI sites of pCSf107 plasmid vector, which carries the CMV IE94 promoter. This fusion protein was designed to remove the signal peptide of hFzd5, resulting in direct fusion of the N-terminus of hFzd5 to the 2xMyc tag. DNA encoding the full length of mouse Wnt3a and EGFP fused Wnt3a(GFP-Wnt3a) was integrated into pCS2 plasmid vector.

### Western blotting and luciferase reporter assay

For detection of proteins in cultured cells and culture supernatant, samples were collected at the time points described in “Cell Culture and Transfection.” To detect Wnt3a-Fzd5 proteins in embryos, the area posterior to the newly formed somite of E8.5 embryos was cut and collected. Samples prepared from two embryos were applied to each lane. SDS-PAGE was carried out according to a standard protocol^65^. Briefly, each sample was mixed with 2x sample buffer [4% SDS, 20% glycerol, 0.001% bromophenol blue and 0.125 M Tris-HCl (pH 6.8)] and heated at 37 °C for 1 h. Samples were electrophoresed using 10% polyacrylamide gels. After electroporation, proteins on the gel were transferred to a polyvinylidene difluoride (PVDF) membrane (Millipore). These membranes were treated overnight at 4 °C with primary antibody (rabbit anti-mouse Wnt3a antibody (polyclonal) prepared by us^66^, 1:500, for Fig. 1h, and mouse anti-mouse Wnt3a antibody (monoclonal) prepared also by us^66^, 1:2 dilution of hybridoma supernatant, for Supplementary Fig. 1a), followed by treatment with secondary antibodies (anti-rabbit-IgG HRP conjugated, Jackson Immuno Research 111-035-144, 1:8000, for Fig. 1h, and goat anti-mouse IgG: HRP conjugated, Promega W402B, 1:3000, for Supplementary for Fig. 1a, b) for 1 h at room temperature. Finally, these proteins were visualized using an Enhanced Chemiluminescent Detection System (Amersham).

Luciferase reporter assay was performed according to the manufacturer’s protocol (Dual-Glo Luciferase Assay System: Promega). Since STF293 cells contain a firefly Luciferase cDNA driven by eight tandem repeats of the TCF binding site, Wnt activity was quantified by monitoring the activity of firefly Luciferase. Renilla luciferase was used as an internal control to compensate for the mosaic nature of gene transfection. The activity of Luciferase was detected using a Luminometer (Turner Designs).

### Generation of *Wnt3a-Fzd5* knock-in mice

In *Wnt3a-Fzd5* knock-in mice, a DNA fragment encoding the MYC-hFZD5 fragment was integrated just before the stop codon in exon4 of the mouse *Wnt3a* gene (Supplementary Fig. 1). The resulting protein expressed from this recombined locus is the same as that expressed in the cell culture experiment described above. To generate this knock-in allele, a pLSODN-3-based plasmid containing a DNA fragment of *myc-Fzd5* was co-injected with plasmids to express gRNA and Cas9 in fertilized eggs. The sequence of the gRNA is as follows: 5′-TTAGGAGCTCTCCTACTTGC-3′. This gRNA was inserted into pX330. Genotyping was carried out by PCR using the following primers:

Wnt3a-Fzd5 5F, 5′-TGGTGCTTATCTGCCATTCC-3′;

Wnt3a-Fzd5 WTF2, 5′-GTCACATGCACCTCAAGTGC-3′;

Wnt3a-Fzd5 7F, 5′-GGTGTGCCAGGAAATCACGG-3′;

Wnt3a-Fzd5 7R, 5′-GGACACCTGCTTGTGGTAGG-3′;

Wnt3a-Fzd5 WTR2, 5′-AGGATCCTTCCTAGCAGTCC-3′;

Wnt3a-Fzd5 4R, 5′-TTTCTACAGTTGACCGGCTC-3′.

The combination of primers used for PCR is shown in Supplementary Fig. 1. Fragments of 2564-bp and 3,338-bp were expected from the 5′-region of wild-type *Wnt3a* and *Wnt3a-Fzd5* alleles, respectively. On the other hand, a 2368-bp fragment and a 3564-bp fragment were expected in the 3′-region of wild-type *Wnt3a* and *Wnt3a-Fzd5* alleles, respectively.

### In situ hybridization

Whole-mount in situ hybridization was performed using digoxigenin-labeled, antisense RNA probes. Briefly, embryos collected at the indicated stages were fixed with 4% paraformadyhyde (PFA) overnight at 4 °C, washed with PBS, and treated with 20 µg/mL of proteinase K for 5 min. These embryos were incubated in hybridization buffer (50% formamide, 5× SSC, 1% SDS, 50 µg/mL tRNA) overnight at 55 °C. The next day, embryos were washed consecutively with 5× SSC, 2× SSC, and Tris-buffered saline with Tween 20 (TBST). Next, embryos were incubated with 1% sheep serum (Sigma) in TBST for 1 h and then treated with a 1:500 dilution of anti-digoxigenin-AP Fab fragments (Roche) overnight at 4 °C. The following day, embryos were washed with TBST and alkaline phosphatase buffer [100 mM NaCl, 100 mM Tris-HCl (pH 9.5), 50 mM MgCl_2_, 0.5% Tween 20], and signals were developed using BM Purple (Roche). Wild-type and mutant embryos were stained for the same period in individual experiments. The following probes, which have been reported previously, were used: mouse *Wnt3a*^67^, mouse *Brachyury*^39^ and mouse *Tbx6*^22,40^, mouse *Uncx4.1*^38^, and human *Fzd5*^68^. To generate a *Sox2* probe, the first exon of mouse *Sox2* was amplified from mouse genomic DNA and cloned to generate an antisense probe.

### Immunofluorescence

Whole-mount immunofluorescence was performed on embryos collected at the indicated stages. These embryos were fixed with 4% PFA overnight at 4 °C, and washed with PBS. Embryos were incubated overnight at 4 °C with the following primary antibodies: rabbit anti-Sox2 (polyclonal, Millipore, AB5603, 1:200) and goat anti-Brachyury (polyclonal, Santacruz, 17745, 1:1000). After washing with PBS, embryos were incubated overnight at 4 °C with DAPI (Dojindo) and following secondary antibodies: donkey anti-rabbit IgG (Alexa fluor 647 conjugated, Invitrogen A31573, 1:500) and donkey anti-goat IgG (Alexa fluor 647 conjugated, Invitrogen A21432, 1:500). Then, embryos were rinsed with PBS again and mounted on 0.8% LMP agarose for observation. Fluorescent images were acquired using an inverted confocal microscope (Leica SP8).

### Retinoic acid treatment

To detect sensitivity to RA signaling in *Wnt3a-Fzd5* homozygotes, we crossed *Wnt3a-Fzd5* heterozygotes. RA dissolved in DMSO was injected into the abdominal cavities of pregnant female mice at 7.5 dpc at a dose equivalent to 6 mg per whole body weight, and embryos were harvested at 8.5 dpc.

### Wnt vis reporter detection

Embryos were harvested at each stage and fixed overnight in 4% PFA. Then, they were stained with DAPI in 1% Triton X-100 solution (1:2000) for several hours or overnight and mounted in 0.8% LMP agarose. Fluorescent images were acquired using an inverted confocal microscope (Leica SP8). The fluorescence of this reporter in the nucleus was measured in individual cells in a photon-counting mode. Epiblast cells in the region lateral to the node were individually analyzed in a single confocal plane. The area of the nuclei in each cell was identified by DAPI staining. GFP intensity in the identified area was determined. For cells with the top 10% GFP intensity in region 2, the relative GFP intensities of cells in contact with those high-GFP cells were summarized.

### Quantification and statistical analysis

Information about statistical details and methods is indicated in the figure legend. Statistical analyses were performed using R for Mac (ver. 4.1.1) and Excel (ver.16.66.1) softwares. Data are expressed as mean ± SD (standard deviation). Differences were assessed for statistical significance as indicated in the figure legends. *P* values <0.05 were considered statistically significant.

### Mathematical model

We considered an ideal situation in which ${L}_{x}\times {L}_{y}$ epiblast cells align on a regular square lattice such that the *x* and *y* axes coincide with the left-right and the anterior-posterior axes (Fig. 7a). Each cell has its Wnt signaling activity as a variable ${W}_{{ij}}$, where *i* and *j* are the indexes of the cell on the *x*–*y* coordinate. The activity ${W}_{{ij}}$ changes in time through the production, autocatalytic reaction, degradation, and intercellular diffusion of Wnt molecules (Fig. 7a), and its time evolution is given by Langevin equation:

1 $${\tau \frac{{{{{{\rm{d}}}}}}}{{{{{{{\rm{d}}}}}}t}}W}_{\!\!ij}=a+\frac{\alpha }{1+{{C}_{{RA}}(\,\,j,t)}^{2}}\frac{{{W}_{{ij}}}^{2}}{{K}^{2}+{{W}_{{ij}}}^{2}}-{W}_{{ij}}+D\mathop{\sum}\limits_{k,k{\prime} \in n.n.}\left({W}_{k,k{\prime} }-{W}_{{ij}}\right)+{\gamma \xi }_{{ij}}(t)$$

where $\tau$ is a constant parameter for determining timescale. The first and second terms on the right side represent the production of Wnt molecules; A constant production (1st term) is described by the parameter $a$, and the autocatalytic reaction (2nd term) is represented by the Hill equation with half saturation concentration *K*. RA-dependent repression of the autocatalytic reaction is described by the pre-factor of the Hill equation with the autocatalysis strength $\alpha$ and RA concentration ${C}_{{RA}}(j,t)$. When RA diffusion does not reach the region of epiblast cells (t < t_RA, e.g., before E.8.0), the value of ${C}_{{RA}}(j,t)$. is set to 0$.\,$After RA diffusion reaches this region $(t \ge {t}_{{RA}}\,{{{{{\rm{e}}}}}}.{{{{{\rm{g}}}}}}.,\,{{{{{\rm{after}}}}}} \; {{{{{\rm{E}}}}}}.8.0),$ ${C}_{{RA}}(j,t)$ creates the following time-independent spatial gradient that increases linearly along y-axis (the anterior-posterior axis) with a slope $\bigtriangleup$:

$${C}_{{RA}}(j,t)={c}_{0}+j\bigtriangleup .$$

The third term in Eq. (1) represents the degradation of Wnt molecules, while the fourth term denotes intercellular diffusion of Wnt molecules with diffusion constant *D*. The summation $\mathop{\sum}\nolimits_{k,{k}^{{\prime} }\in n.n.}(\,)\,$indicates the summation with respect to the nearest-neighbor cells around ${W}_{{ij}}$ (i.e., ${W}_{i\pm 1,j}$ and ${W}_{i,j\pm 1}$). The last term in Eq.(1) denotes the noise term: $\gamma$ and ${\xi }_{{ij}}(t)$ indicate the noise strength and independent Gaussian white noise with zero mean and variance $\langle {{\xi }_{ij}}(t){{\xi}_{i ^\prime j ^\prime}} (t ^{\prime} )\rangle=\delta \left(t-{t}{{^\prime}}\right){{\delta }_{i,i ^\prime}}{{\delta }_{j,\, j ^\prime}},$ respectively. Cell division events are also considered a stochastic Poisson process in the model. Each cell divides randomly at a constant rate $\lambda$. By cell division, surrounding cells are pushed stochastically toward either the left (in the negative direction of the x-axis with probability 1/4), right (along the positive direction of x-axis with probability 1/4) or upward (along the positive direction of y-axis with probability 1/2). For instance, when the left is chosen in the division of cell with $x=i$ and $y=j$, all cells with $x < i$ and $y=j$ move toward the left. The extruded cell outside the ${L}_{x}\times {L}_{y}$ lattice is removed. The ${W}_{{ij}}$ value of the dividing cell is inherited by daughter cells.

Equation (1) is numerically solved by the Euler–Maruyama method with $\triangle t={10}^{-5}$, while the presence or absence of a cell division event is determined at each time step following the probability ${\lambda L}_{x}{L}_{y}\Delta t$. The time unit is normalized so that $\lambda=1$, while the length scale is normalized by cell length (the lattice size). The diffusion constant *D* is set to *D* = 0.2 for paracrine (+) cells (Fig. 7b, d and e) and D = *0* for paracrine (-) cells (Fig. 7c and f). The production rate of Wnt molecules was chosen as $a=0.025$ for paracrine ($\pm$) cells (Fig. 7b, c, e and f) and $a=0.0$ for paracrine ($+$)-production ($\downarrow$) cells (Fig.7d). The parameter ${c}_{0}$ that represents basal RA concentration for $t\ge {t}_{{RA}}$ was chosen as$\,{c}_{0}=0.1$ for RA(-) situation (Fig.7b-d) and$\,{c}_{0}=0.15$ for RA(+) situation (Fig.7e and f). For other parameters, the following values were used: ${L}_{x}={L}_{y}=50$,$\,\alpha=0.8,{K}=0.38,\,\tau=0.02,\,\Delta=0.005,\,\gamma=0.01.$

### Reporting summary

Further information on research design is available in the Nature Portfolio Reporting Summary linked to this article.

## Supplementary information

Supplementary Information

Description of Additional Supplementary Files

Supplementary Movie 1

Supplementary Movie 2

Supplementary Movie 3

Supplementary Movie 4

Supplementary Movie 5

Reporting Summary

## Data Availability

All other data that support the findings of this study are provided in the article or Supplementary Information/Source Data file. Source data are provided with this paper.

## Source data

Source Data
