# Supplementary material for: Intercellular exchange of Wnt ligands reduces cell population heterogeneity during embryogenesis
Source: Nat Commun. 2023 Apr 6;14:1924. doi: 10.1038/s41467-023-37350-x (PMC10079677; doi:10.1038/s41467-023-37350-x)
Supplement: Supplementary file 1 — Supplementary Information [file 41467_2023_37350_MOESM1_ESM.pdf]

Supplementary Information

Intercellular exchange of Wnt ligands reduces cell population heterogeneity during embryogenesis.

Yudai Hatakeyama, Nen Saito, Yusuke Mii, Ritsuko Takada, Takuma Shinozuka, Tatsuya Takemoto, Honda Naoki, & Shinji Takada*

*Coresponding author: e-mail: stakada@nibb.ac.jp and nensaito@hiroshima-u.ac.jp

This PDF file includes:

Supplementary Figure 1-12

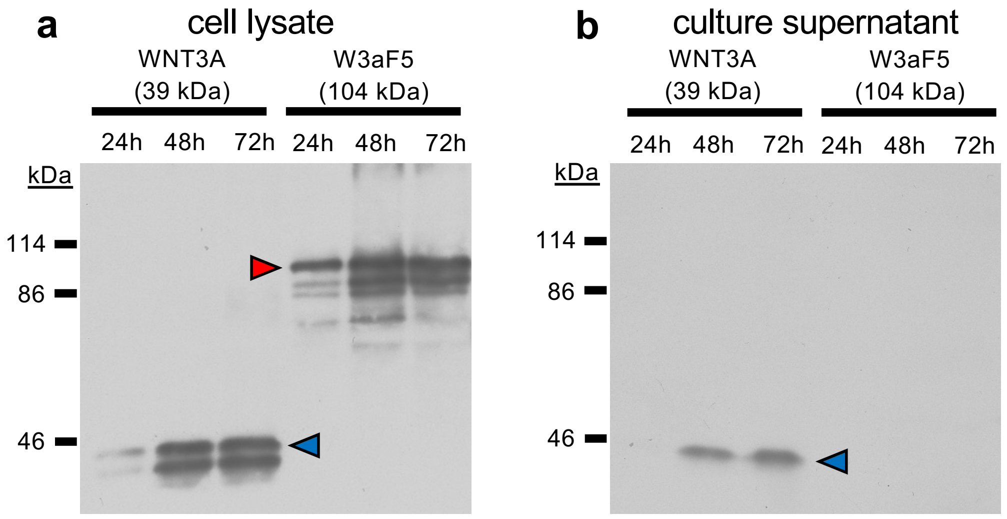

Supplementary Fig. 1. WNT3A-FZD5 was not detected in culture supernatant

Western blotting analysis of cell lysate (**a**) and culture supernatant (**b**) prepared from WNT3A and WNT3A-FZD5 expressing HEK293T cells, 24, 48, and 72 h after transfection. While the expression level of WNT3A-FZD5 was similar to WNT3A in the cell lysate, WNT3A-FZD5 was not detectable in culture supernatant. Red and blue arrowheads indicate bands corresponding to the predicted molecular weights of WNT3A-FZD5 and WNT3A, respectively. The photographs shown in (**a**) and (**b**) are representative of two independent experiments with similar results.

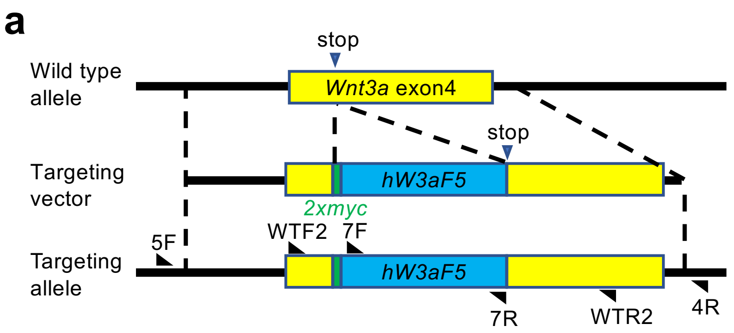

b

Combination of primer	wt allele	Targeting allele
5F/7R	-	3338 bp
7F/4R	-	3564 bp
5F/WTR2	2546 bp	(4304 bp)
4R/WTF2	2368 bp	(4126 bp)

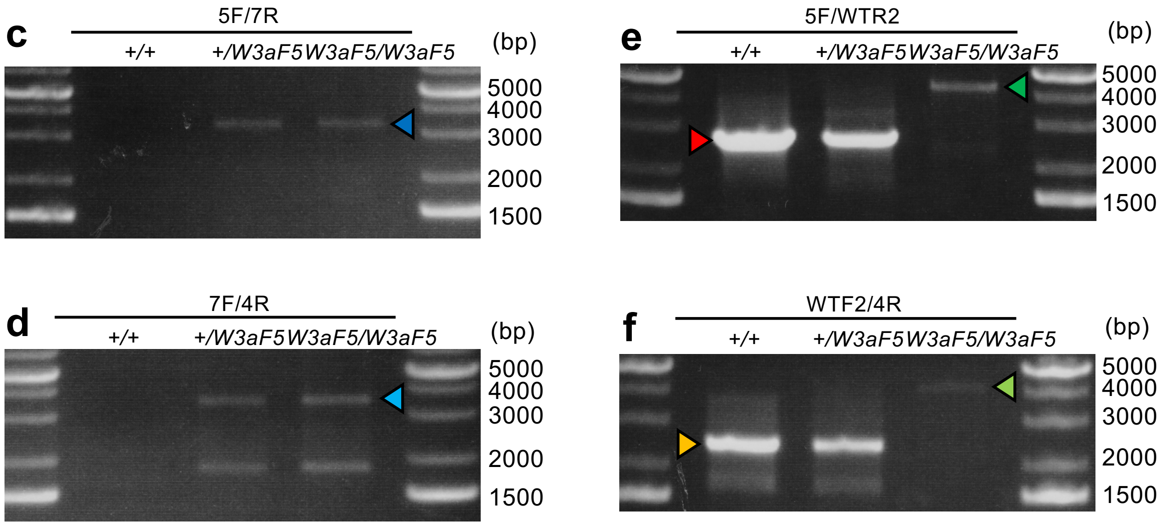

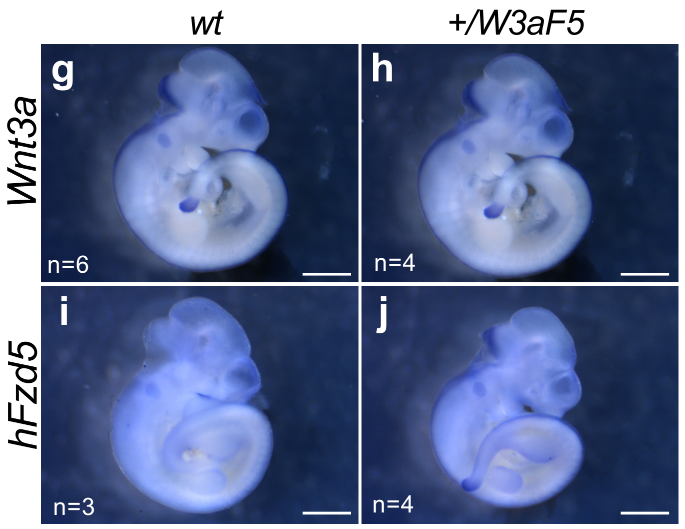

k

	% of individuals		
stage	*wt*	*+/W3aF5*	*W3aF5/W3aF5*
E10.5	8 (17.0)	29 (61.7)	10 (21.3)
E12.5	9 (18.0)	28 (56.0)	13 (26.0)
after birth	10 (28.6)	25 (71.4)	0 (0)

Supplementary Fig. 2. Generation of Wnt3a-Fz5 knock-in mice
(**a**-**f**) Generation the *Wnt3a-Fzd5* knock-in allele. A schematic diagram indicates the mouse *Wnt3a* locus and the *Wnt3a-Fzd5* knock-in allele is shown in (**a**). In the knock-in allele, human Frizzled5 (blue) fused with 2 myc tags (green) is inserted at the C-terminus of mouse Wnt3a. The knock-in event was confirmed by PCR analysis using the primer sets indicated in **b**. The results of PCR analyses are shown (**c**-**f**). Primer sets are indicated on the upper side of each figure. Band sizes indicated by colored arrowheads correspond to the predicted sizes shown in **b**. The photographs shown in (**c**) to (**f**) are representative of at least ten independent experiments with similar results. (**g**-**j**) Whole image of *wt* (**g**, **i**) and *Wnt3a*$^{+/Fzd5}$ (**h**, **j**) embryos at E10.5 hybridized with *Wnt3a* (**g**, **h**) or *hFzd5* (**i**, **j**) probes. Magnified images of posterior bodies and dorsal views of these embryos are shown in Fig. 1d-g. Numbers of stained embryos are indicated by "n=" in the images. Scale bars: 1 mm. (**k**) The proportion of individuals of each genotype during embryonic development and immediately after birth.

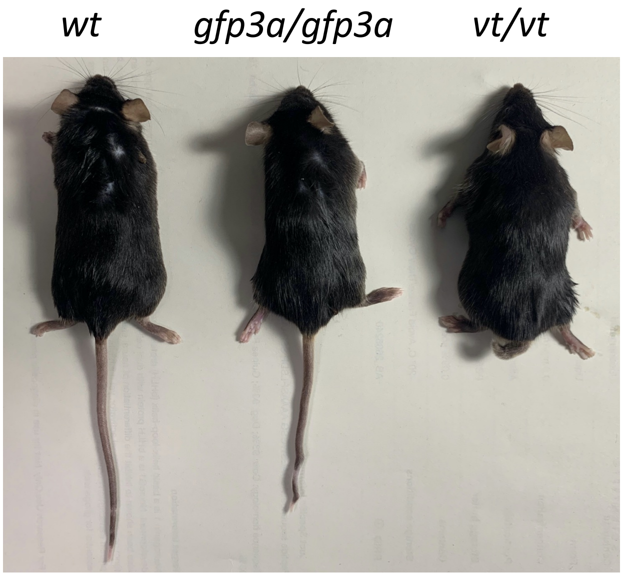

	Short tail/total (%)
WT	0/20 (0%)
gfp3a/gfp3a	11/21 (52.3%)
vt/vt	27/27 (100%)

Supplementary Fig. 3. Tail morphology of Wnt3a mutant mice
Tail morphology of adult *wt*, $Wnt3a^{gfp3a/gfp3a}$, and $Wnt3a^{vt/vt}$ mice were compared. Percentage of mice exhibiting short tail is shown in the table.

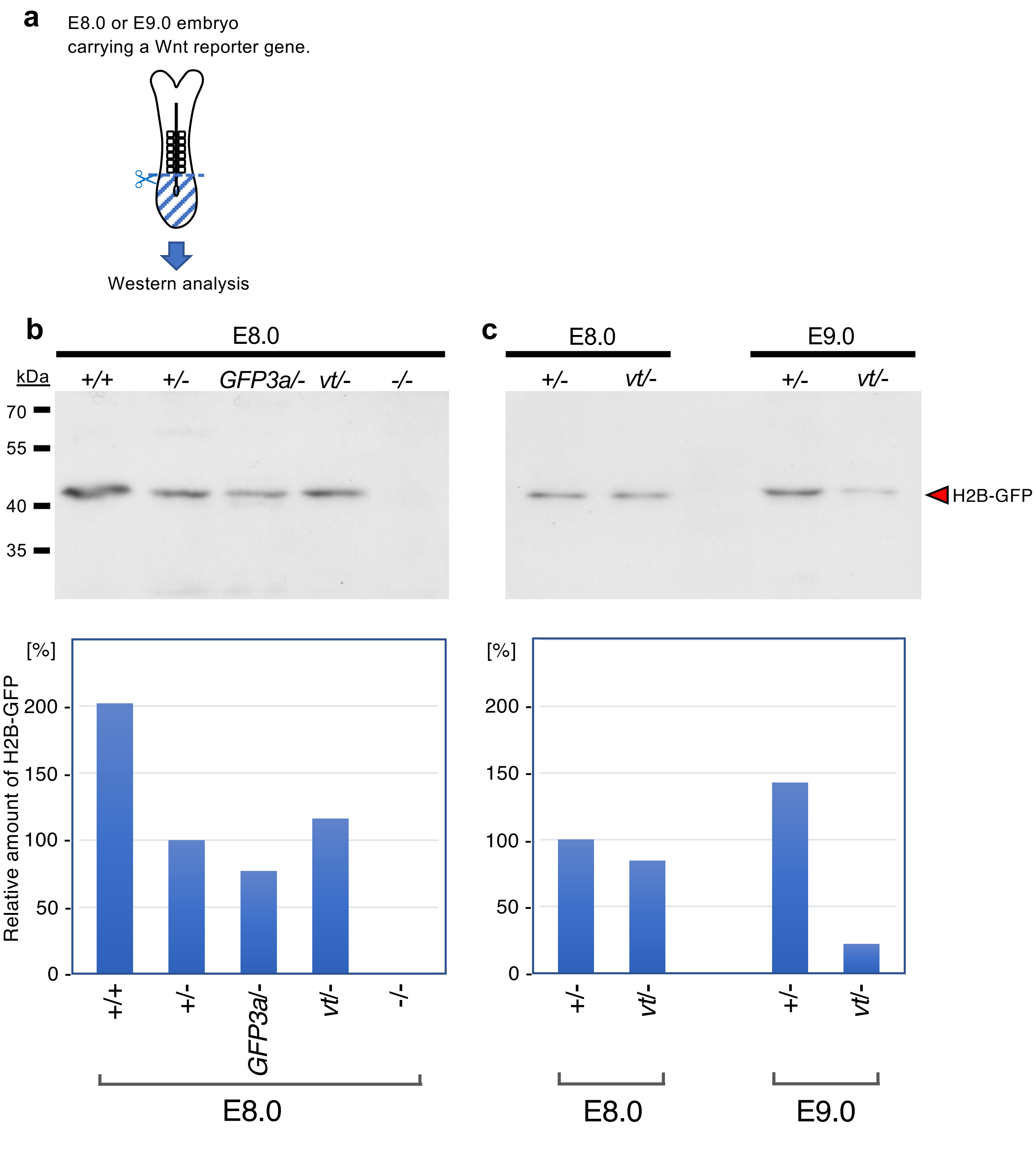

Supplementary Fig. 4. Wnt signaling activity in the tailbud region of Wnt3a mutant embryos
To monitor Wnt signaling activity in epiblast/tailbud region, posterior region shown in (**a**) was collected. Wnt signaling activity was monitored using Histone 2B (H2B)-GFP-reporter gene, expression of which is specifically activated by Wnt signaling[44]. Wnt mutant mice were crossed with mice carrying the GFP-reporter gene and crossed in several generations. Wnt activity in the epiblast/tailbud region of resulting offspring embryos carrying one copy of the reporter gene was examined at E8.0 (**b**, **c**) and E9.0 (**c**) by Western blotting using anti-GFP antibody. The intensity of bands corresponding to H2B-GFP was quantitated using NIH image. The results indicate that Wnt activity in GFP-Wnt3a/- embryos is 70% of that of *Wnt3a*$^{+/-}$ embryos at E8.0 (**b**). On the other hand, Wnt activity in *vt/-* embryos abruptly reduces at E9.0 (**c**), consistent with *in situ* hybridization (Supplementary Fig. 7). The photographs and data shown in (**b**) and (**c**) are representative of two independent experiments with similar results.

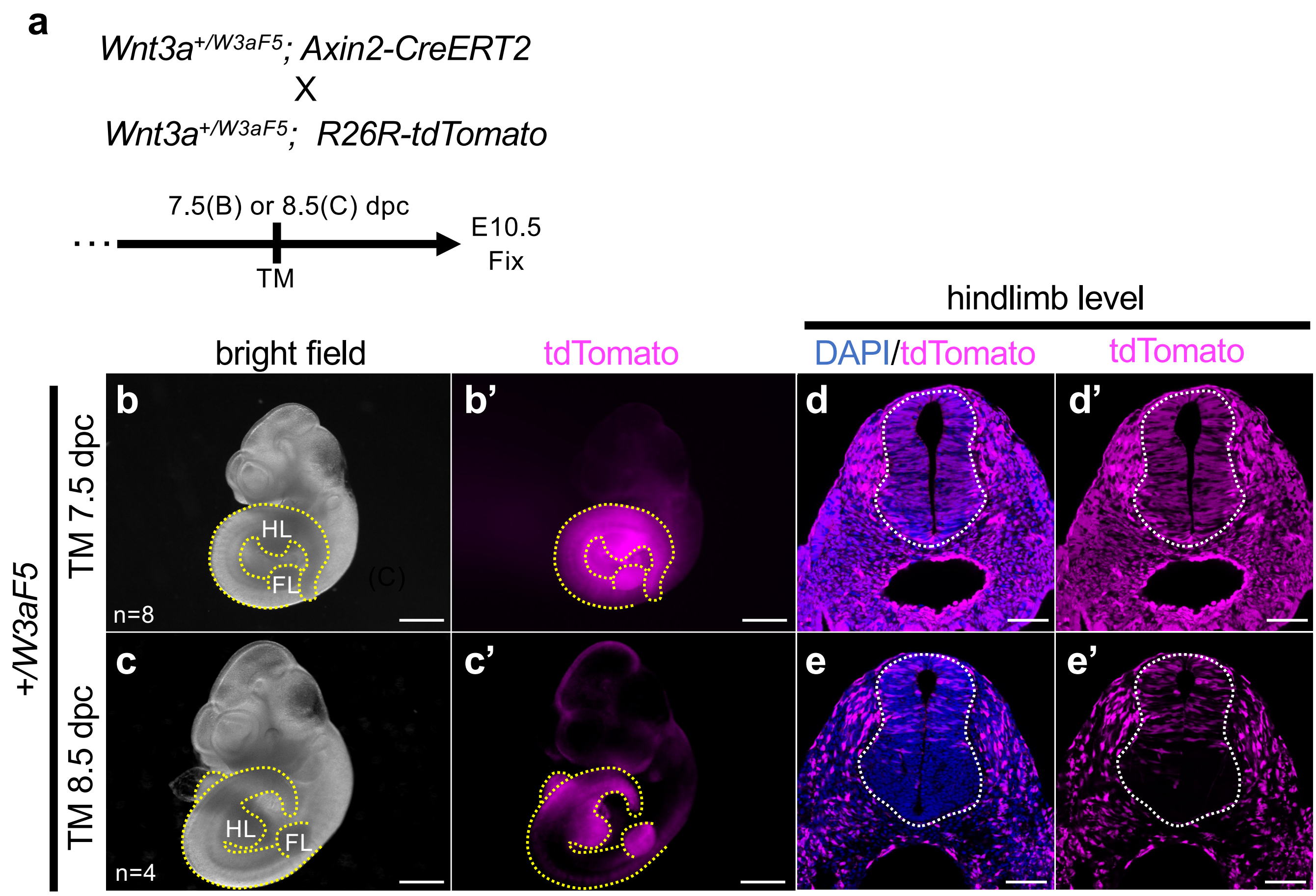

Supplementary Fig. 5. The source of ventral neural cells loses Wnt signaling after E7.5 during development of *Wnt3a-Fzd5* heterozygous embryos

Experimental procedure is shown in (**a**). Cells activated by Wnt signaling and monitored by *Axin2-CreERT2* expression, were eternally labeled with *tdTomato* expression. Tamoxifen (TM) was injected to pregnant females at 7.5 dpc (**b**, **d**) or 8.5 dpc (**c**, **e**) and embryos were fixed at E10.5. Whole-mount bright field images (**b**, **c**) and tdTomato staining are also indicated (**b'**, **c'**). Distribution of tdTomato-labeled cells at the posterior hindlimb level in *Wnt3a*$^{+/Fzd5}$ embryos at E10.5 (**d**, **e**). Numbers of stained embryos are indicated by "n=" in (**b**) and (**c**). Scale bars: 100 µm

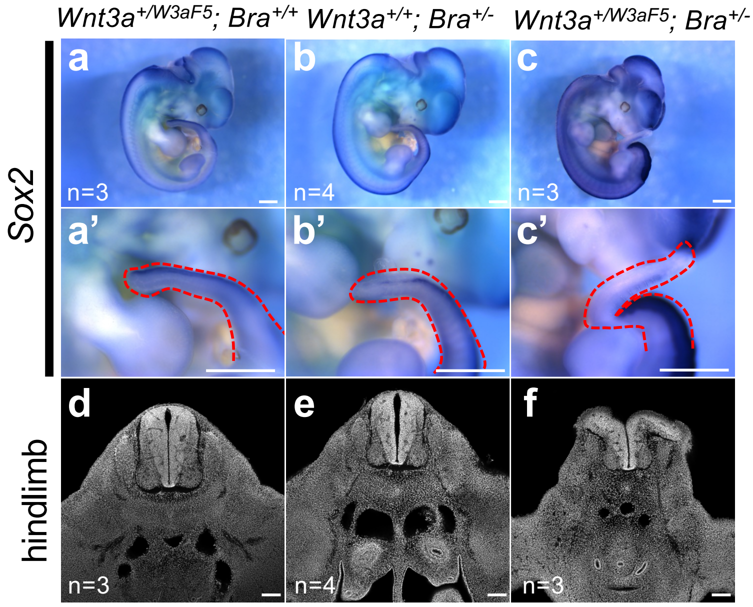

Supplementary Fig. 6. Synergistic effect of Wnt3a-Frizzled and Bra on the posterior development of body axis elongation

(**a**-**c**) *Wnt3a*$^{+/Fzd5}$*; Bra*$^{+/+}$(**a**), *Wnt3a*$^{+/+}$*; Bra* $^{+/-}$(**b**) and *Wnt3a*$^{+/Fzd5}$*; Bra*$^{+/-}$(**c**) embryos stained by whole-mount *in situ* hybridization using *Sox2* probe are shown. Embryos were fixed at E11.5. **a'**, **b'**, and **c'** are magnified images of **a**, **b**, and **c**, respectively. (**d**-**f**) Transverse sections of the neural tube of *Wnt3a*$^{+/Fzd5}$*; Bra*$^{+/+}$(**d**), *Wnt3a*$^{+/+}$*; Bra* $^{+/-}$(**e**) and *Wnt3a*$^{+/Fzd5}$*; Bra*$^{+/-}$(**f**) embryos at E11.5. Note that *Wnt3a-Fzd5* and *Bra* compound heterozygous embryos (*Wnt3a*$^{+/Fzd5}$*; Bra*$^{+/-}$(**c**, **f**)) impair the posterior development of body axis elongation while embryos heterozygous for either of them appear normal. Numbers of stained embryos are indicated by "n=" in the images. Scale bars: 1 mm (**a**-**c**, **a'**-**c'**), 100 μm (**d**-**f**).

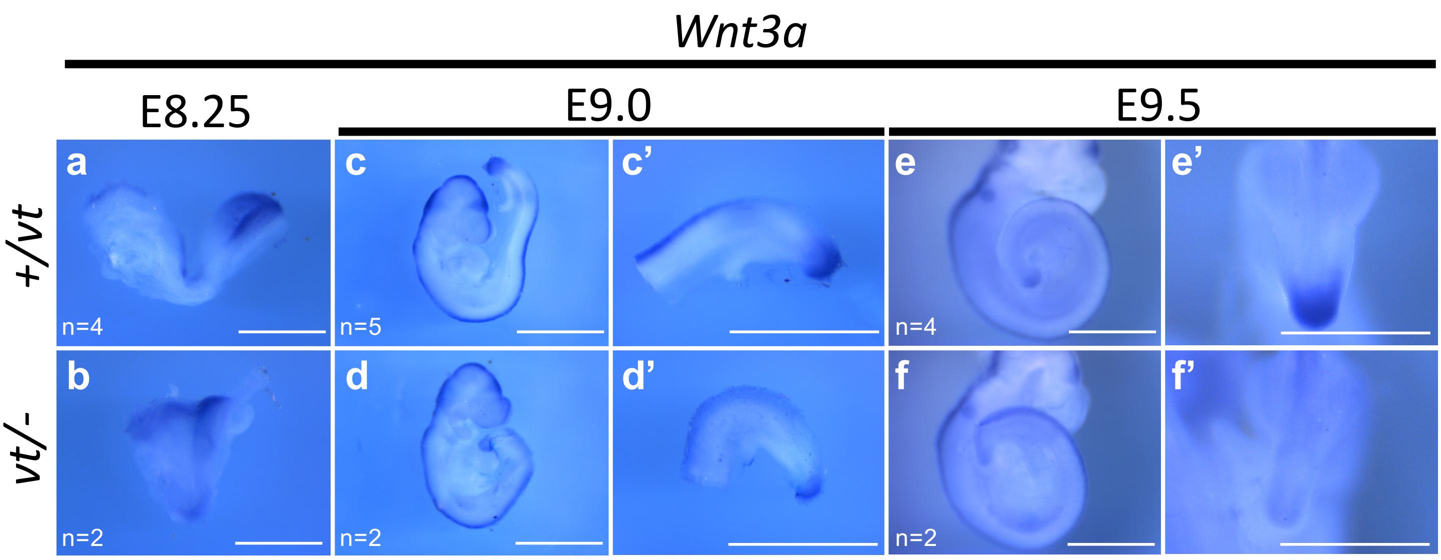

Supplementary Fig. 7. Wnt3a expression in *Wnt3a*$^{vt/-}$ embryos

Wnt3a expression was detected by whole-mount *in situ* hybridization of *Wnt3a*$^{+/vt}$ (**a**, **c**, **e**) and *Wnt3a*$^{vt/-}$ (**b**, **d**, **f**) embryos at E8.25 (**a**, **b**), E9.0 (**c**, **d**), and E9.5 (**e**, **f**). Magnified images of the posterior region of each embryo are indicated (**c'**-**f'**). In *Wnt3a*$^{vt/-}$ embryos, *Wnt3a* expression decreases in the tailbud after E9.0 and is rarely detected in E9.5. Numbers of stained embryos are indicated by "n=" in the images. Scale bars: 1 mm.

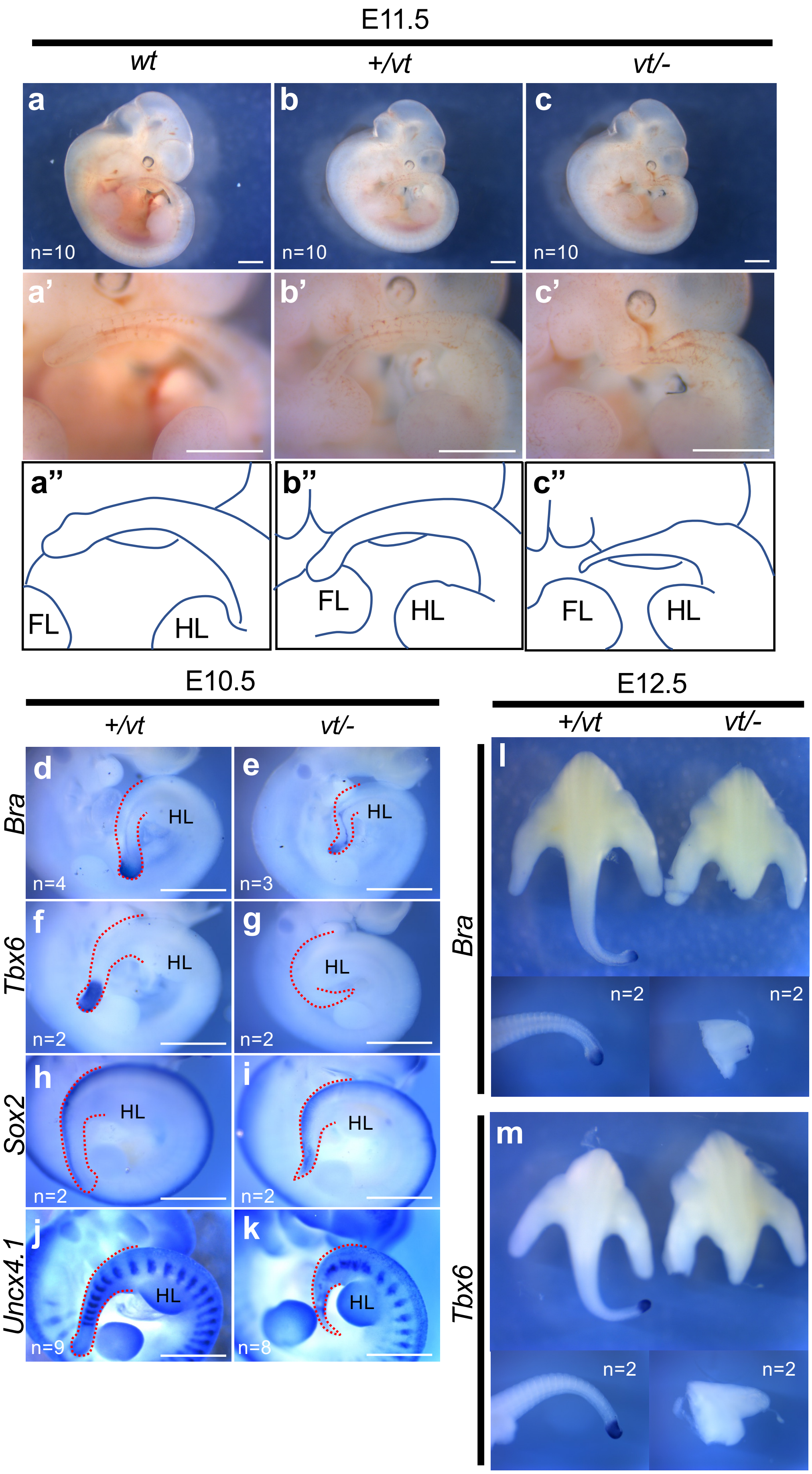
E11.5
wt
+/vt
vt/-
a
b
c
n=10
n=10
n=10
a'
b'
c'
a''
b''
c''
FL
HL
FL
HL
FL
HL
E10.5
+/vt
vt/-
E12.5
+/vt
vt/-
Bra
Tbx6
Sox2
Uncx4.1
d
e
f
g
h
i
j
k
l
m
HL
n=4
n=3
n=2
n=2
n=2
n=2
n=9
n=8
Bra
Tbx6
n=2
n=2
n=2
n=2

Supplementary Fig. 8. Characteristics of *Wnt3a*$^{vt/-}$ embryos

(**a**-**c**) Lateral views of *wt* (**a**), *Wnt3a*$^{+/vt}$ (**b**) and *Wnt3a*$^{vt/-}$ (**c**) embryos at the E11.5. **a'**, **b'**, and **c'** are magnified images of **a**, **b**, and **c**, respectively. **a''**, **b''**, and **c''** are drawings of the images of **a'**, **b'**, and **c'**, respectively. (**d**-**k**) Expression of mesoderm and neural marker genes in *Wnt3a*$^{+/vt}$ (**d**, **f**, **h**, **j**) and *Wnt3a*$^{vt/-}$ (**e**, **g**, **i**, **k**) embryos at E10.5. Whole-mount *in situ* hybridization was carried out using probes of *Bra* (**d**, **e**), *Tbx6* (**f**, **g**), *Uncx4.1* (**h**, **i**), and *Sox2* (**j**, **k**). Red dotted lines indicate tail regions. (**l**, **m**) Whole-mount *in situ* hybridization of *Wnt3a*$^{+/vt}$ and *Wnt3a*$^{vt/-}$ embryos at E12.5 using *Bra*(**l**) and *Tbx6*(**m**) probes. In contrast to *Wnt3a-Fzd5* homozygous embryos, expression of *Bra* nad *Tbx6* is not detectable in *Wnt3a*$^{vt/-}$ embryos. Numbers of stained embryos are indicated by "n=" in the images. HL: hindlimb. Numbers of stained embryos are indicated by "n=" in the images. Scale bars: 1 mm.

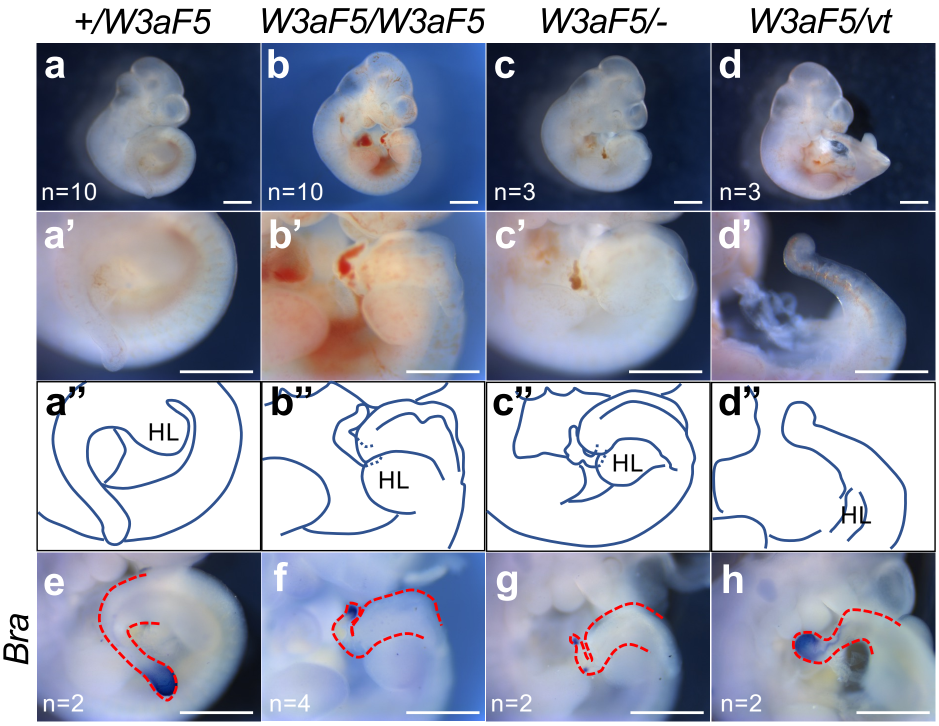

Supplementary Fig. 9. The phenotype of *Wnt3a-Fzd5* homozygous embryos can be rescued, depending on intercellular signaling of Wnt3a

(**a-d**) Sagittal views of *Wnt3a*$^{+/Fzd5}$ (**a**, **a'**, **a''**), *Wnt3a*$^{Fzd5/Fzd5}$ (**b**, **b'**, **b''**), *Wnt3a*$^{Fzd5/-}$ (**c**, **c'**, **c''**) and *Wnt3a*$^{Fzd5/vt}$ (**d**, **d'**, **d''**) embryos at the E10.5. **a'**, **b'**, **c'**, and **d'** are magnified images of **a**, **b**, **c**, and **d**, respectively. **a''**, **b''**, **c''**, and **d''** are drawings of the images of **a'**, **b'**, **c'**, and **d'**, respectively. (**e-h**) Whole-mount *in situ* hybridization of *Wnt3a*$^{+/Fzd5}$ (**e**), *Wnt3a*$^{Fzd5/Fzd5}$ (**f**), *Wnt3a*$^{Fzd5/-}$ (**g**) and *Wnt3a*$^{Fzd5/vt}$ (**h**) embryos at E10.5 with *Bra* probe. Red dotted lines indicate the edge of the body posterior to the hindlimb. Numbers of stained embryos are indicated by "n=" in the images. Scale bars: 1 mm.

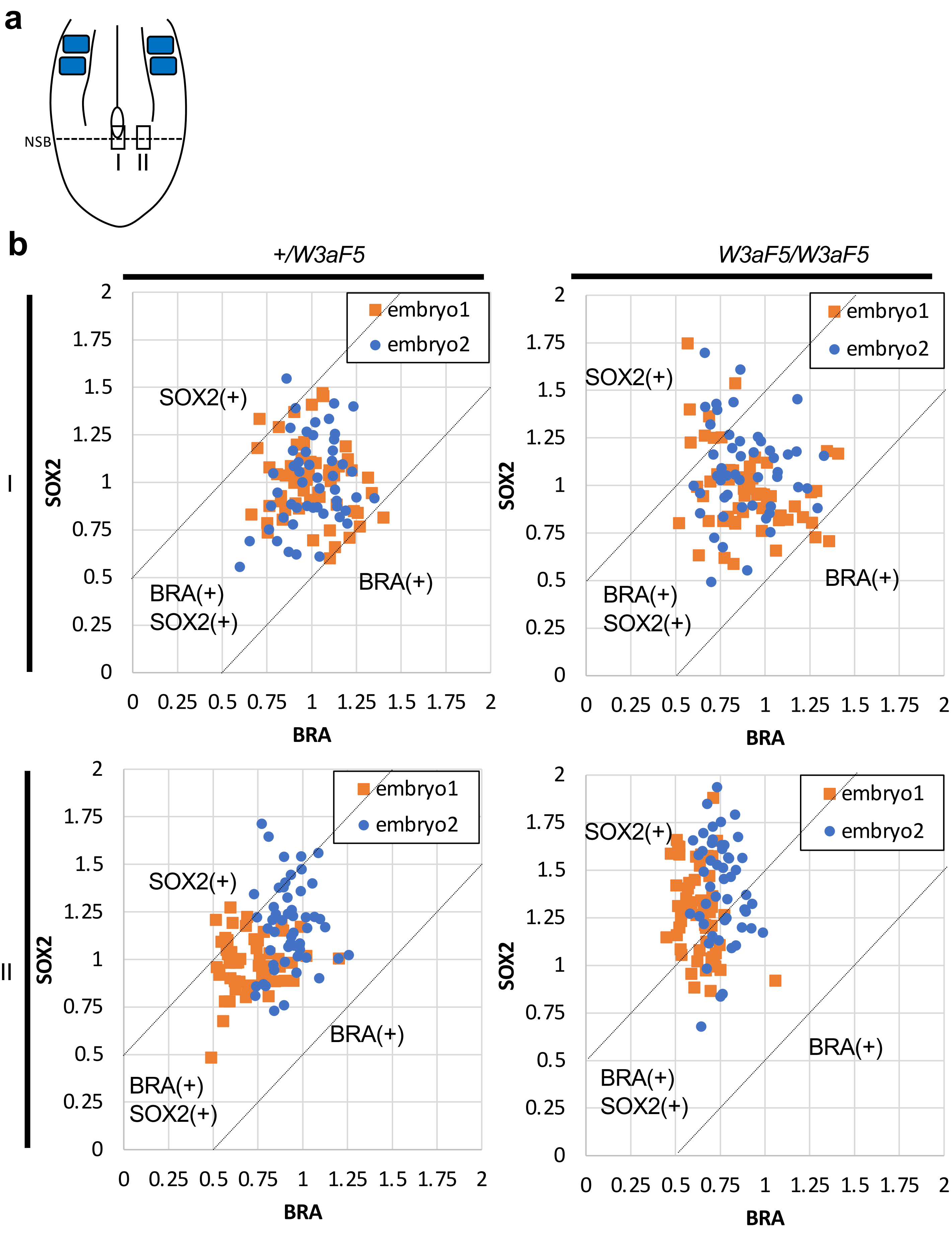
a
NSB
I
II
b
+/W3aF5
W3aF5/W3aF5
I
II
SOX2
BRA
embryo1
embryo2
SOX2(+)
BRA(+)
BRA(+)
SOX2(+)

Supplementary Fig. 10. Summary plots of Bra and Sox2 signal intensities examined by immunohistochemistry (**a**) Schematic figure showing the area examined. (**b**) Summary plots of BRA and SOX2 signal intensities in medial (I) and lateral (II) areas at the node-streak border in *Wnt3a-Fzd5* heterozygous and homozygous embryos. Two embryos were examined for each genotype. Measurements for each cell are plotted according to levels of BRA (x-axis) and SOX2 (y-axis). Levels of BRA and SOX2 in each cell were normalized by the average of levels of BRA and SOX2 level in the medial area of *Wnt3a-Fzd5* heterozygous embryos. Cells located between the two dashed lines were defined as BRA and SOX2 double-positive cells.

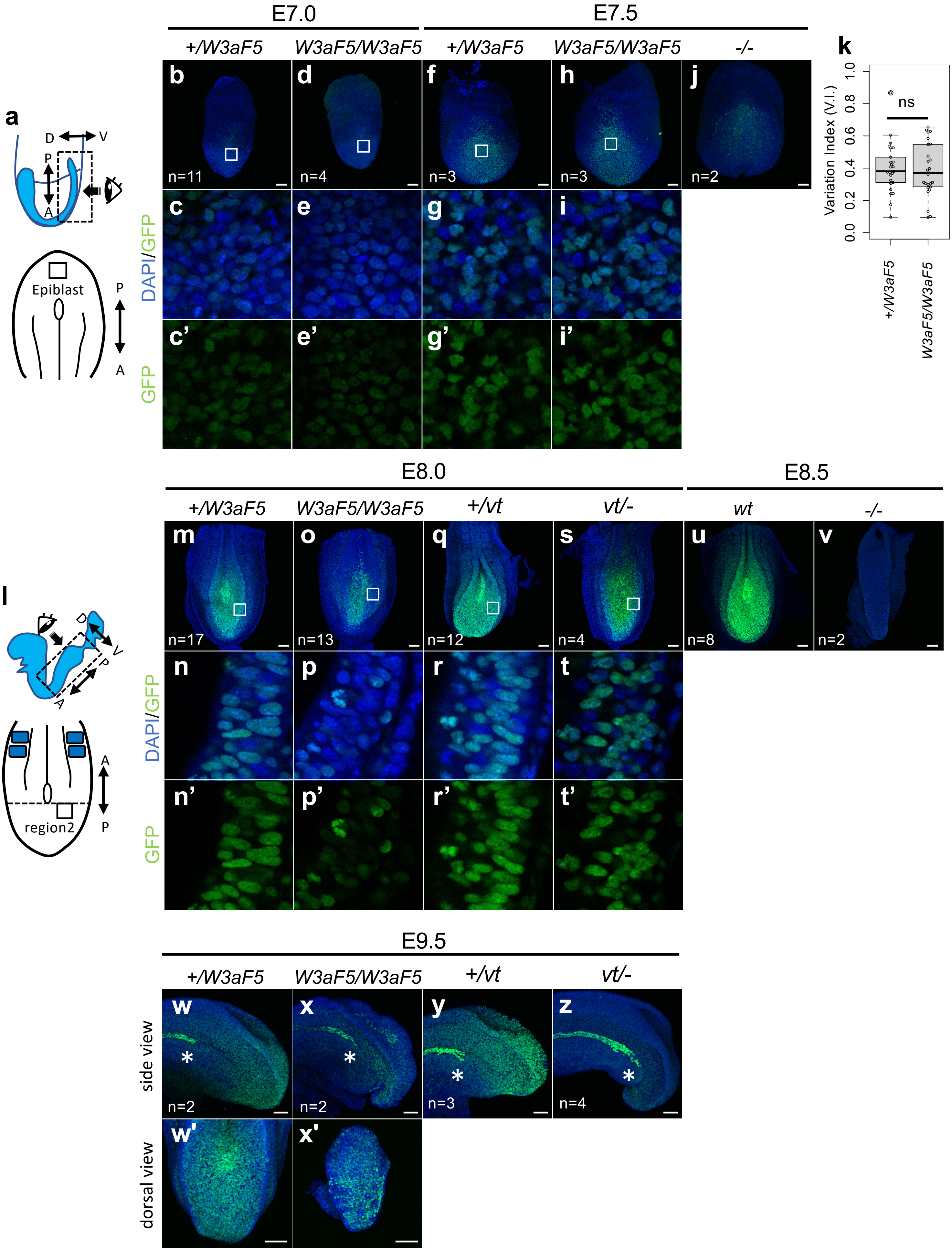
E7.0
E7.5
+/W3aF5
W3aF5/W3aF5
+/W3aF5
W3aF5/W3aF5
-/-
a
D
V
P
A
Epiblast
P
A
b
d
f
h
j
n=11
n=4
n=3
n=3
n=2
c
e
g
i
DAPI/GFP
c'
e'
g'
i'
GFP
k
ns
Variation Index (V.I.)
0.0
0.2
0.4
0.6
0.8
1.0
+/W3aF5
W3aF5/W3aF5
E8.0
E8.5
+/W3aF5
W3aF5/W3aF5
+/vt
vt/-
wt
-/-
l
D
V
P
A
A
P
region2
m
o
q
s
u
v
n=17
n=13
n=12
n=4
n=8
n=2
n
p
r
t
DAPI/GFP
n'
p'
r'
t'
GFP
E9.5
+/W3aF5
W3aF5/W3aF5
+/vt
vt/-
side view
w
x
y
z
n=2
n=2
n=3
n=4
dorsal view
w'
x'

Supplementary Fig. 11. Wnt signaling in the epiblast cell population of Wnt3a knock-out embryos
This is a supplemental figure to Fig. 5. Wnt signaling activity in individual epiblast cells was visualized using mouse embryos carrying an GFP-reporter gene, expression of which is specifically activated by Wnt signaling[44]. The observation schemes of embryos at cylinder stage (E7.0 and E7.5) and somite stage (E8.0 and E8.5) are shown in (**a**) and (**l**), respectively. An eye mark indicates the direction of observation. Wnt signaling activity was monitored in *Wnt3a-Fzd5* heterozygous (**b**, **c**, **f**, **g**, **m**, **n, w**) and homozygous (**d**, **e**, **h**, **i**, **o**, **p**, **x**) embryos at E7.0 (**b**-**e**), E7.5 (**f**-**i**), E8.0 (**m**-**p**), and E9.5 (**w**, **x**). Wnt signaling activity was also visualized in *+/vt* (**q**, **r**, **y**) and *vt/-* (**s**, **t**, **z**) embryos at E8.0 (**q**-**t**) and E9.5 (**y**, **z**). In embryos shown in (**b**), (**d**), (**f**), (**h**), (**m**), (**o**), (**q**), and (**s**), magnified images of the areas indicated by boxes are also shown in (**c**), (**e**), (**g**), (**i**), (**n**), (**p**), (**r**) and (**t**), respectively. In embryos shown in (**w**) and (**x**), dorsal view of the posterior tip regions are also shown in (**w'**) and (**x**'), respectively. Note that Wnt signaling activity is not obviously changed in *Wnt3a-Fzd5* homozygous embryos at E7.0 (**b**, **d**) or at E7.5 (**f**, **h**). On the other hand, this activity is severely decreased, but still some Wnt-positive cells remain, in *Wnt3a-Fzd5* homozygous embryos at E 9.5. The degree of difference, defined as "variation index"(Fig.5o), is examined for *Wnt3a-Fzd5* heterozygous (**g**) and homozygous (**i**) at E7.5. Each dot in (**k**) indicates variation index around one of cells in the top 10% of Wnt activity. Wnt signaling activity was also monitored in wild type (**u**) and Wnt3a knock-out (**j**, **v**) and embryos at E7.5 (**j**) and E8.5 (**u**, **v**). Also, note that Wnt signaling is drastically reduced at E7.5 (**j**) and completely lost at E8.5 (**v**) in *Wnt3a* null embryos, suggesting that Wnt activity at and after E8.5 epiblast is dependent on only Wnt3a ligand. Magnified images were taken at a single confocal plane while others were processed by maximum intensity projection. Numbers of stained embryos are indicated by "n=" in the images. Scale bars: 100 μm. In (**k**), the middle, upper, and lower box lines represent the maximum, minimum, median and two quartiles of values in each group and whiskers indicate highest and lowest values no greater than 1.5 × interquartile range. Differences were assessed for statistical significance using a two-sided Student's t-test; ns (not statistically significant, P=0.76). The star in the E9.5 shows the nephric duct.

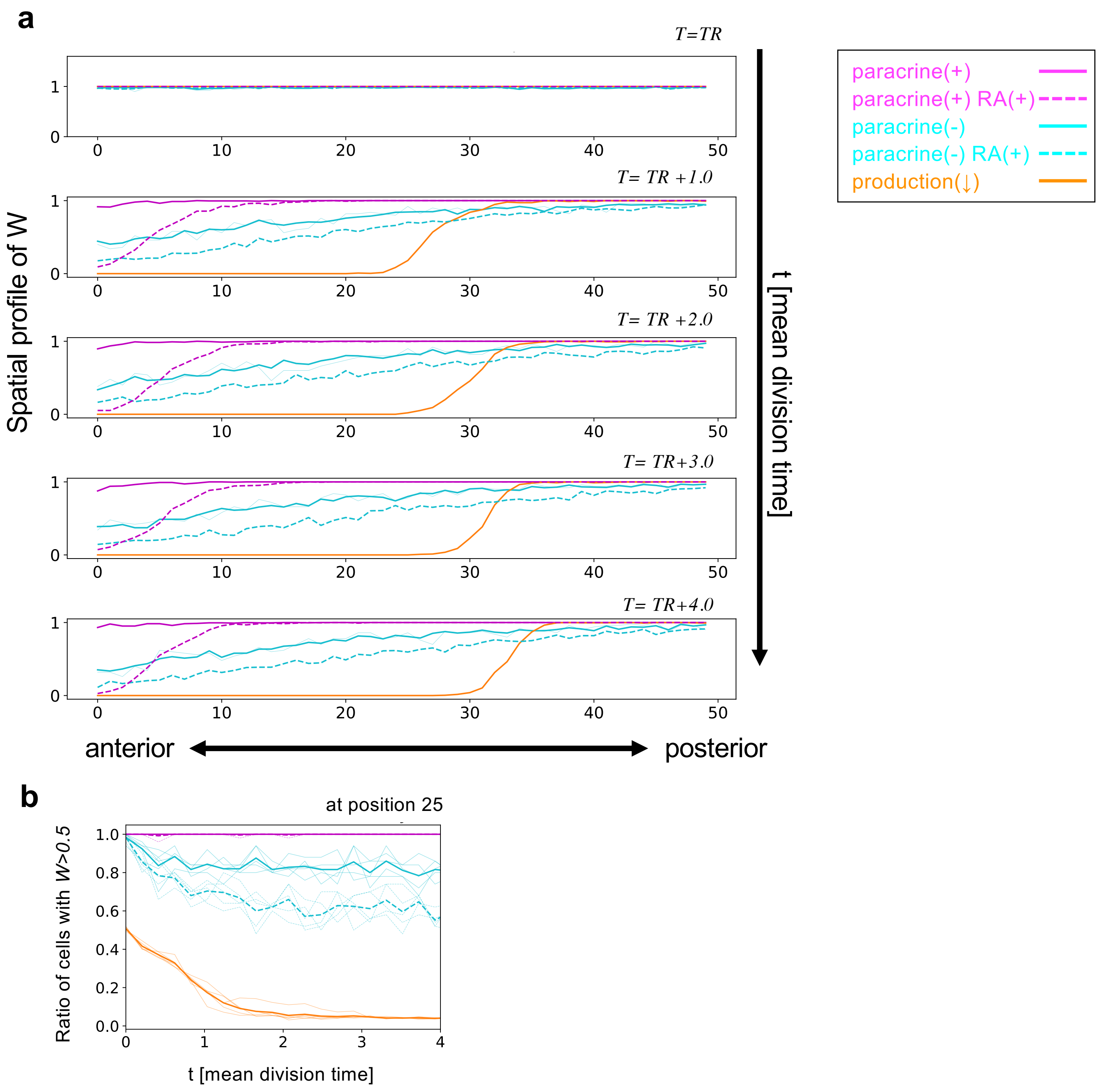

Supplementary Fig. 12. Temporal changes of the spatial profile of Wnt-positive cells as simulated by our mathematical model

(**a**) Spatial profiles of the proportion of Wnt-positive cells (>50% of maximum activity) in the virtual space are indicated at T=TR, TR+1, TR+2, TR+3 and TR+4, where the time unit is normalized by mean division time after supply of RA from the anterior side. Spatial profiles under combined conditions with and without Wnt-mediated intercellular communication and with and without uniformly supplied RA are shown. Also shown is the spatial profile under Wnt-mediated intercellular communication and a reduced rate of Wnt production. In Fig. 7G, the time course of the proportion of Wnt-positive cells at the same spatial level (y=25) is summarized in a single graph. A and P indicate anterior and posterior, respectively. (**b**) Time course of Wnt-positive cells in a virtual sheet of cells. The time course of the proportion of Wnt-positive cells (>50% of maximum activity) at the same spatial level (y=35 in b-d) along the anterior-posterior axis in a virtual sheet of cells is shown. Orange and blue lines indicate the result with and without the paracrine function of Wnt, respectively. A green line indicates the result obtained in which the Wnt production rate is reduced in the presence of the paracrine function of Wnt. Solid and dashed lines indicate results obtained in the absence and presence of uniformly supplied RA, respectively.
