# Supplementary material for: Intercellular exchange of Wnt ligands reduces cell population heterogeneity during embryogenesis
Source: Nat Commun. 2023 Apr 6;14:1924. doi: 10.1038/s41467-023-37350-x (PMC10079677; doi:10.1038/s41467-023-37350-x)
Supplement: Supplementary file 2 — Description of Additional Supplementary Files [file 41467_2023_37350_MOESM2_ESM.pdf]

Description of Additional Supplementary Files

File name: Supplementary Movie 1
Description: Simulation of the time course of Wnt activity in the hypothetical epiblast in the presence of intercellular exchange of Wnt ligands.

File name: Supplementary Movie 2
Description: Simulation of the time course of Wnt activity in the hypothetical epiblast in the absence of intercellular exchange of Wnt ligands.

File name: Supplementary Movie 3
Description: Simulation of the time course of Wnt activity in the hypothetical epiblast in the presence of intercellular exchange of Wnt ligands, but reduced Wnt production.

File name: Supplementary Movie 4
Description: Simulation of the time course of Wnt activity in the hypothetical epiblast with uniform addition of RA in the presence of intercellular exchange of Wnt ligands.

File name: Supplementary Movie 5
Description: Simulation of the time course of Wnt activity in the hypothetical epiblast with uniform addition of RA in the absence of intercellular exchange of Wnt ligands.
