# Supplementary material for: Intercellular exchange of Wnt ligands reduces cell population heterogeneity during embryogenesis
Source: Nat Commun. 2023 Apr 6;14:1924. doi: 10.1038/s41467-023-37350-x (PMC10079677; doi:10.1038/s41467-023-37350-x)
Supplement: Supplementary file 8 — Reporting Summary [file 41467_2023_37350_MOESM8_ESM.pdf]

nature portfolio

Corresponding author(s): Shinji Takada

Last updated by author(s): 2023/03/03

Reporting Summary

Nature Portfolio wishes to improve the reproducibility of the work that we publish. This form provides structure for consistency and transparency in reporting. For further information on Nature Portfolio policies, see our Editorial Policies and the Editorial Policy Checklist.

Statistics

For all statistical analyses, confirm that the following items are present in the figure legend, table legend, main text, or Methods section.

n/a	Confirmed	
☐	☒	The exact sample size (*n*) for each experimental group/condition, given as a discrete number and unit of measurement
☐	☒	A statement on whether measurements were taken from distinct samples or whether the same sample was measured repeatedly
☐	☒	The statistical test(s) used AND whether they are one- or two-sided<br>*Only common tests should be described solely by name; describe more complex techniques in the Methods section.*
☒	☐	A description of all covariates tested
☒	☐	A description of any assumptions or corrections, such as tests of normality and adjustment for multiple comparisons
☐	☒	A full description of the statistical parameters including central tendency (e.g. means) or other basic estimates (e.g. regression coefficient) AND variation (e.g. standard deviation) or associated estimates of uncertainty (e.g. confidence intervals)
☐	☒	For null hypothesis testing, the test statistic (e.g. *F*, *t*, *r*) with confidence intervals, effect sizes, degrees of freedom and *P* value noted<br>*Give P values as exact values whenever suitable.*
☒	☐	For Bayesian analysis, information on the choice of priors and Markov chain Monte Carlo settings
☒	☐	For hierarchical and complex designs, identification of the appropriate level for tests and full reporting of outcomes
☒	☐	Estimates of effect sizes (e.g. Cohen's *d*, Pearson's *r*), indicating how they were calculated

Our web collection on statistics for biologists contains articles on many of the points above.

Software and code

Policy information about availability of computer code

Data collection	N/A
Data analysis	Image J (ver. 2.3.0) was used for analyzing western blot images. R for Mac (ver. 4.1.1) and Excel (ver.16.66.1) were used for statistical analysis. Leica amplification suite X (ver.3.1.5) was used for imaging analysis.

For manuscripts utilizing custom algorithms or software that are central to the research but not yet described in published literature, software must be made available to editors and reviewers. We strongly encourage code deposition in a community repository (e.g. GitHub). See the Nature Portfolio guidelines for submitting code & software for further information.

Data

Policy information about availability of data

All manuscripts must include a data availability statement. This statement should provide the following information, where applicable:

- Accession codes, unique identifiers, or web links for publicly available datasets
- A description of any restrictions on data availability
- For clinical datasets or third party data, please ensure that the statement adheres to our policy

All raw data used in this study are included in Source Data File.

Human research participants

Policy information about studies involving human research participants and Sex and Gender in Research.

Reporting on sex and gender	N/A
Population characteristics	N/A
Recruitment	N/A
Ethics oversight	N/A

Note that full information on the approval of the study protocol must also be provided in the manuscript.

Field-specific reporting

Please select the one below that is the best fit for your research. If you are not sure, read the appropriate sections before making your selection.

☒ Life sciences ☐ Behavioural & social sciences ☐ Ecological, evolutionary & environmental sciences

For a reference copy of the document with all sections, see nature.com/documents/nr-reporting-summary-flat.pdf

Life sciences study design

All studies must disclose on these points even when the disclosure is negative.

Sample size	No sample size calculation was performed. Detailed n number are provided in the figures or figure legends. In studies with mouse embryos, the sample size was determined according to previous studies using similar analyses (Garriock et al. Development 2015, Shinozuka et al. Development 2019).
Data exclusions	No data exclusion except Fig. 5. In these experiments, embryos that failed to acquire fluorescent images were excluded.
Replication	Indicated in figure legends. Basically, the analyses were repeated at least three times (sometimes twice). All experimental data were consistent in all replicates.
Randomization	In experiments shown in Fig.1-4, embryos with the same genotype and similar embryonic stage were randomly assigned to groups prior to initiation of experiments. In experiments shown in Fig. 5 and 6, fluorescence images of all embryos in pups were obtained before genotype of each embryo was determined.
Blinding	No blinding was used because all experiments were conducted and analyzed by one person. Genotyping of each embryo was performed automatically, regardless of the results of experiments with each embryo.

Reporting for specific materials, systems and methods

We require information from authors about some types of materials, experimental systems and methods used in many studies. Here, indicate whether each material, system or method listed is relevant to your study. If you are not sure if a list item applies to your research, read the appropriate section before selecting a response.

Materials & experimental systems

n/a	Involved in the study	
☐	☒	Antibodies
☐	☒	Eukaryotic cell lines
☒	☐	Palaeontology and archaeology
☐	☒	Animals and other organisms
☒	☐	Clinical data
☒	☐	Dual use research of concern

Methods

n/a	Involved in the study	
☒	☐	ChIP-seq
☒	☐	Flow cytometry
☒	☐	MRI-based neuroimaging

Antibodies

Antibodies used	Antibodies used for Western blotting: mouse anti-Wnt3a antibody (generated by ourselves: (Takada et al., Dev. Cell, 2006, 11, 791-801) and goat anti-mouse IgG:HRP conjugated (Promega W402B). Antibodies used for immunostaining of embryos: rabbit anti-

	Sox2 (polyclonal, Millipore, AB5603, 1:200), goat anti-Brachyury (polyclonal, Santacruz, 17745, 1:1000), donkey anti-rabbit IgG (Alexa fluor 647 conjugated, Invitrogen A31573, 1:500),and donkey anti-goat IgG (Alexa fluor 647 conjugated, Invitrogen A21432, 1:500).
Validation	Mouse anti-Wnt3a antibody was validated in Takada et al., Dev. Cell, 2006 by Westerni blotting with Wnt3a expressing and parental L cell. Anti-Sox2 and anti-Bra antibodies was validated by Western blotting and expression patterns in mouse embryos at E8.5 and E11.5.

Eukaryotic cell lines

Policy information about cell lines and Sex and Gender in Research

Cell line source(s)	HEK 293T and STF293 were gifted by Dr. Takeichi (RIKEN) and Dr. Tsukiyama (Tsukiyama et al. Mol Cell Biol . 2015, 35, 2007-2023), respectively.
Authentication	STF293 was validated by STR profiling.
Mycoplasma contamination	Cell lines were tested negative for Mycoplasma contamination.
Commonly misidentified lines (See ICLAC register)	No commonly misidentified lines were used.

Animals and other research organisms

Policy information about studies involving animals; ARRIVE guidelines recommended for reporting animal research, and Sex and Gender in Research

Laboratory animals	Mice were maintained in a light- and temperature-controlled room using a 12 h:12 h light:dark cycle at 21±2℃. The humidity in the breeding room was maintained between 45% and 65%. Wnt3a null and vt mice were gifted by Dr. Andrew McMahon. GFP-Wnt3a knock-in mice were previously generated by ST. Brachyury null mutant mice were distributed by RIKEN BRC (RBRC00113 : C3H/HeSn-Ttf/+tf). Wnt vis reporter mice were previously generated by TT.C57BL/6N mice were obtained by Japan SLC and CLEA-Japan or generated in house. In all experiments, mice between 8 and 60 weeks old were used for mating to obtain embryos. Tamoxifen was dissolved in corn oil and injected into the abdominal cavity of mice.
Wild animals	No wild animals were used in the study.
Reporting on sex	The sex of all embryos used in this study was not examined.
Field-collected samples	No field collected samples were used in the study.
Ethics oversight	Animal care and experiments were performed in accordance with guidelines for animal experimentation of the National Institutes for Natural Sciences. All animal experiments were approved by the Animal Research Committee of National Institutes for Natural Sciences.

Note that full information on the approval of the study protocol must also be provided in the manuscript.
